# Surrogate Scattering Matrix‐Guided Inverse Design of Nanophotonic Neural Networks

**DOI:** 10.1002/nap2.70302

**Published:** 2026-09-23

**Authors:** Azka Maula Iskandar Muda, Uğur Teğin

**Affiliations:** ^1^ Department of Electrical and Electronics Engineering Koç University İstanbul Türkiye

**Keywords:** adjoint optimization, image classification, inverse design, nanophotonic media, optical computing, photonic neural networks

## Abstract

Optical computing offers a route to address the growing computational demands of machine learning, with inverse‐designed nanophotonic media providing a compact path to passive optical operators. Training such devices end to end remains expensive because each geometry update requires full‐wave forward and adjoint evaluations. We introduce a surrogate‐guided inverse‐design framework that separates task learning from electromagnetic realization. Classification is first solved in matrix space using a passive complex operator with bounded singular values, and the resulting target is transferred to a fabrication‐aware freeform device through complex transmission matching and reflection suppression. Because the realization target is fixed, the electromagnetic work per update is determined by the input and output port counts and is independent of training‐set size. A locality‐constrained router composed with a fixed evanescent mixing stage extends the approach to 16‐port transformations. Under the same physical initialization, solver, fabrication map, and 550‐solve budget, operator matching reached 98.34% test accuracy on MedNIST, compared with 89.50% for direct geometry‐to‐task training. On RSSCN7, the combined optical surrogate reached 48.21%, compared with 38.57% for a PCA‐16 ridge classifier. Three Yin–Yang realizations reached 91.44±2.02%. These results establish surrogate‐guided operator matching for compact, fabrication‐aware photonic neural networks.

## Graphical abstract

Surrogate‐guided inverse design separates task learning from electromagnetic realization by transferring a trained passive operator to a fabrication‐aware nanophotonic structure. The electromagnetic cost of each update depends on port count rather than the dataset size. Validated with freeform and evanescent‐waveguide devices to implement MedNIST, RSSCN7, and Yin–Yang classifiers, this method enables a more efficient way of inverse designing nanophotonics neural networks. The illustration was created using OpenAI ImageGen via ChatGPT (accessed 24 August 2026).

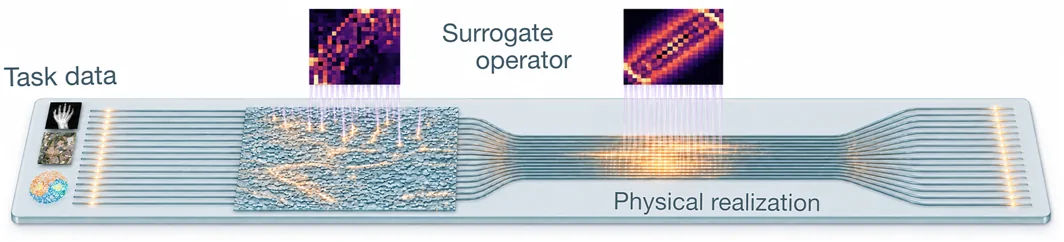

## 1 Introduction

Deep neural networks now drive a broad range of applications, from image recognition to natural language processing. Training these models demands growing amounts of compute, and digital electronics is approaching its efficiency limits. Optical computing offers a hardware route to address this bottleneck. Photonic neural networks perform matrix–vector multiplication at the speed of light with passive components. Several architectures have been demonstrated, including coherent Mach–Zehnder meshes [1, 2, 3], diffractive deep networks [4], phase‐change photonic tensor cores [5], and on‐chip photonic classifiers [6]. The unitary factors in programmable meshes follow the Reck and Clements decompositions [7, 8]; the bounded singular values in Equation (1) extend this representation to passive nonunitary operators. Recent reviews frame these systems as physical neural networks whose capacity is set by the interplay between trainable parameters and hardware physics [9, 10, 11, 12].

Wave‐based analog computing established scattering‐space operator design through metamaterial mathematical operations [13], inverse‐designed equation solvers [14], parallel operators in one structure [15], and inverse‐designed structures combined with reconfigurable couplers [16]. Inverse‐designed nanophotonic media offer a compact route to optical linear operators. The entire passive device is represented as a continuous permittivity distribution and optimized so that its scattering matrix implements the desired map [17, 18]. Gradients are computed with the adjoint method [19]. Khoram et al. showed that freeform nanophotonic media can perform neural inference [20]. Nikkhah et al. experimentally realized vector–matrix multiplication in low‐index‐contrast structures [21]. Zhao et al. and Sved et al. subsequently increased the computational density and scale of these devices [22, 23]. Fabrication‐aware maps, such as conic filtering and subpixel‐smoothed projection [24], impose minimum‐feature constraints during optimization.

Training an inverse‐designed photonic classifier end to end remains expensive because each geometry update requires full‐wave forward and adjoint evaluations. For a linear optical device, field responses can be reused across samples by superposition. The relevant electromagnetic solve count is set by the excited input and adjoint output ports and is independent of dataset minibatch size.

Here, we decouple these roles with a two‐stage surrogate workflow. In the first stage, the trainable optical block is optimized directly in matrix space using a singular‐value‐bounded parameterization. The resulting operator $T^{⋆}$ is then transferred to a fabrication‐aware freeform device through an adjoint problem. The realization objective is a Frobenius‐norm residual between the simulated transmission matrix and $T^{⋆}$, together with a reflection penalty. Because $T^{⋆}$ is fixed, one update uses $N_{\text{in}}+N_{\text{out}}$ source solves and is independent of the training‐set size. The task‐trained target and the local‐router/fixed‐mixing factorization distinguish this method from established prescribed‐operator matching.

Our second contribution addresses a layout bottleneck in inverse‐designed routers. Routing power between ports separated across the device width increases the required propagation distance along the routing axis. We train a banded router through a differentiable power‐concentration term while retaining every complex matrix entry as a trainable variable. The router is then composed with a fixed downstream evanescent‐coupling region that supplies global mixing [25, 26].

We validate the framework on three tasks. For MedNIST [27], operator matching reaches 98.34% test accuracy under the same 550‐solve budget for which direct geometry‐to‐task optimization reaches 89.50%. For RSSCN7 [28], the combined optical surrogate reaches 48.21%, compared with 38.57% for a PCA‐16 ridge classifier. The Yin–Yang dataset [29] tests the same framework with intensity detection and a digital readout, reaching 91.44±2.02% across three physical initializations. A schematic of the workflow is shown in Figure 1.

**FIGURE 1:**
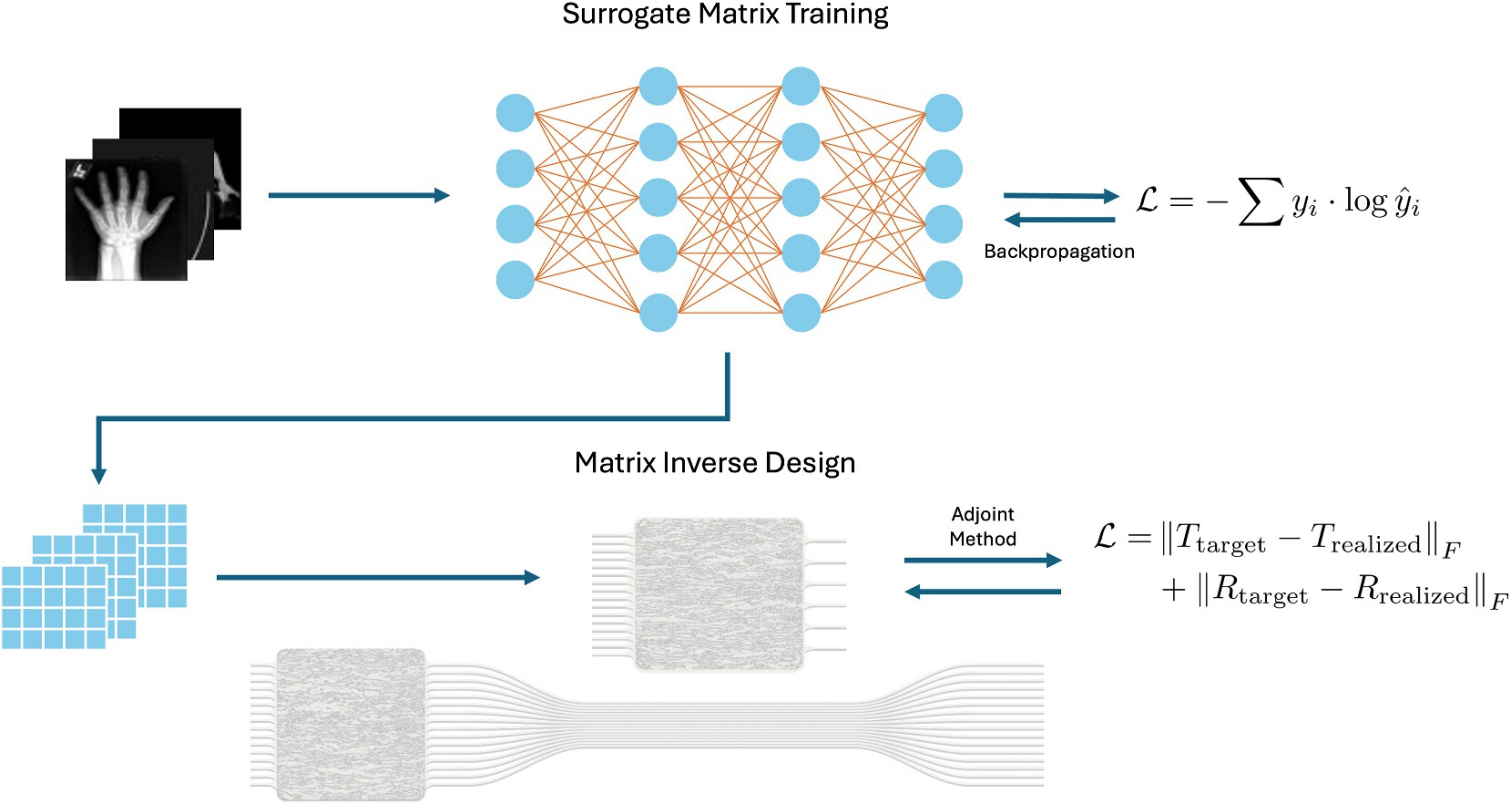
Surrogate matrix‐based inverse design of an optical neural network. In the surrogate stage (top), a passive complex matrix is trained directly on the classification task with cross‐entropy loss. In the realization stage (bottom), the adjoint method is used to match the transmission and reflection blocks of a fabrication‐aware freeform device to the surrogate target via a Frobenius‐norm residual.

## 2 Materials and Methods

The workflow has two stages. First, we solve the learning task in a low‐cost surrogate space by representing the trainable optical block directly as a passive matrix. We then transfer the selected surrogate operator to a fabrication‐aware nanophotonic device through adjoint inverse design. This separation removes dataset batching from the expensive electromagnetic loop and lets the full‐wave solver focus on matching a target scattering response.

### 2.1 Surrogate Operator Model

Let

$$D={\left\{\left(z_{b},y_{b}\right)\right\}}_{b=1}^{B}$$

denote the dataset. We first map each sample z to a reduced feature vector through dimensionality reduction,

$$x=P\left(z\right)\in R^{N_{\mathrm{in}}},$$

and then optically encode it as

$$a=\phi \left(x\right)\in C^{N_{\text{in}}}.$$

The local trainable optical stage is modeled as a passive linear operator

$$M\in C^{N_{\text{out}}\times N_{\text{in}}},$$

parameterized by

(1) $$M=U\;\Sigma \left(s\right)\;V^{†}.$$

here $V\in C^{N_{\text{in}}\times N_{\text{in}}}$ and $U\in C^{N_{\text{out}}\times N_{\text{out}}}$ are unitary matrices acting on the input and output spaces, respectively. We generate these factors from learnable skew‐Hermitian parameters, which preserves unitarity throughout optimization. The matrix $\Sigma \left(s\right)\in R^{N_{\text{out}}\times N_{\text{in}}}$ is rectangular. Its $r=\min\left(N_{\text{out}},N_{\text{in}}\right)$ principal‐diagonal entries are collected in the real singular‐value vector $s\in R^{r}$. In this factorization, U and V represent basis changes, whereas s captures channel‐wise attenuation. We constrain the singular values elementwise as $s_{i}\in \left[s_{\min},s_{\max}\right]$ with $0\leq s_{\min}\leq s_{\max}\leq 1$, which directly enforces passivity and insertion‐loss bounds.

Bounding every singular value by one prevents the target matrix from amplifying any input field. This no‐gain condition is necessary for a passive device, but it does not prove that a finite device can realize the target. To explain the additional restrictions, we write the complete response for excitation from either side as

(2) $$S=\left(\begin{array}{cc} R_{L} & T_{\text{RL}}\\ T_{\text{LR}} & R_{R} \end{array}\right).$$

here, $T_{\text{LR}}$ is the forward transmission optimized in this work, $T_{\text{RL}}$ is transmission in the reverse direction, and $R_{L}$ and $R_{R}$ are the two reflection blocks. Reciprocity requires $S=S^{T}$, which gives $R_{L}=R_{L}^{T}$, $R_{R}=R_{R}^{T}$, and $T_{\text{RL}}=T_{\text{LR}}^{T}$. The forward and reverse responses therefore cannot be chosen independently. Passivity requires $S^{†}S\;⪯\;I$ and therefore

(3) $$R_{L}^{†}R_{L}+T_{\text{LR}}^{†}T_{\text{LR}}\;⪯\;I$$

for excitation from the left. For any coherent input, this inequality states that power returned to the input ports plus power delivered to the monitored output ports cannot exceed the incident power. The remainder is radiated, absorbed, or coupled to modes outside the monitored port basis. The desired transmission and low reflection must therefore be produced simultaneously by the same structure.

The bounded‐SVD parameterization enforces only the no‐gain condition on the forward target. The physical device must additionally obey reciprocity while using fixed port positions, a finite footprint, and the filtered and projected dielectric patterns allowed by the fabrication map. These restrictions make the physically accessible set smaller than the set of all no‐gain matrices. If the target lies outside that accessible set, no design in the chosen device class can reduce the transmission residual to zero. A nonzero plateau is therefore consistent with a physical realization gap, although the nonconvex optimization may also retain error from a local solution.

A fixed downstream optical stage with compatible dimensions is represented by a linear map O. The optical field after the passive stack is

u=OMa,

and the measured optical features are given by the componentwise readout map

$$\Psi \left(u\right)=|u{|}^{2}.$$

Together, these definitions cover both all‐optical and cascaded classifiers. For an all‐optical system, O=I. In chained systems, O denotes a fixed downstream photonic operator, such as the evanescent‐coupling stage used in RSSCN7. Blocks described by full bidirectional scattering matrices are cascaded using the Redheffer star product [30], not by direct matrix multiplication. The reported RSSCN7 model uses the forward transmission product OM because its fixed stage is represented by a forward coupled‐mode transmission matrix.

Surrogate training optimizes the passive operator through

(4) $$\min_{U,V,s}\;\frac{1}{B}\sum_{b=1}^{B}L_{\text{task}}\;\left(\Psi \left(OMa_{b}\right),y_{b}\right)\;+\;\sum_{k}\lambda_{k}\;R_{k}\left(U,V,s\right),$$

where $M=U\Sigma \left(s\right)V^{†}$ and $R_{k}$ denote optional regularizers on the matrix or on its factors. This stage uses matrix products and elementary nonlinearities and requires no electromagnetic simulation.

We consider two task formulations. For all‐optical classification, the optical intensities themselves serve as class logits,

(5) $$\ell_{\text{AO}}\left(M\right)=-\frac{1}{B}\sum_{b=1}^{B}\log\frac{\exp\left(I_{b,y_{b}}\right)}{\sum_{c}\;\exp\left(I_{b,c}\right)},\;I_{b}=|OMa_{b}{|}^{2}.$$

For readout‐based classification, the optical device acts as a feature extractor, and a linear head is applied to the detected intensities. If $I_{b}=|OMa_{b}{|}^{2}$, then the logits are

$$g_{b}=W^{⊤}I_{b}+b,$$

followed by the same cross‐entropy loss. Throughout this work, MedNIST is treated primarily as an all‐optical problem, whereas RSSCN7 is evaluated after composition with a fixed downstream optical stage and a linear readout.

From a computational standpoint, the advantage of the surrogate stage is straightforward. If $C_{\text{LA}}$ denotes the cost of one surrogate update and $C_{\text{EM}}$ the cost of one electromagnetic realization step, then typically $C_{\text{LA}}\ll C_{\text{EM}}$. The two‐stage workflow assigns task learning to the inexpensive surrogate regime and reserves full‐wave optimization for a shorter operator‐matching stage. Once a target operator $T^{⋆}$ is fixed, the expensive stage matches scattering residuals using label‐free adjoint sources [20, 22, 23].

### 2.2 Fabrication‐Aware Inverse Design

Once surrogate training is complete, the selected operator $M^{⋆}$ defines the local transmission target,

$$T^{⋆}=M^{⋆}.$$

During inverse design, only the freeform design region is optimized; the input and output waveguides, tapers, and any optional fixed evanescent‐coupling region remain fixed. We denote the latent design variables by

$$\theta_{\text{raw}}\in {\left[0,1\right]}^{H\times W},$$

and pass through a fabrication‐aware map before simulation,

(6) $$\theta_{\text{filt}}=F_{r_{f}}\left(\theta_{\text{raw}}\right),\;\theta_{\text{sim}}=P_{\beta ,\eta ,\rho }\left(\theta_{\text{filt}}\right).$$

Within this map, $F_{r_{f}}$ is a conic filter with a 200‐nm radius, and $P_{\beta ,\eta ,\rho }$ is Tidy3D's differentiable smoothed projection with β=50 and η=0.5. The 50‐nm parameter grid is clipped to the physical density interval after each optimizer step. Rounded‐corner pixels outside the active design mask are fixed at zero in the parameters, gradient, and both Adam moments. The erosion–dilation penalty is disabled through Update 5, increases from 0.08 at Update 6 to 0.8 at Update 15, and remains 0.8 thereafter. We use the subpixel‐smoothed projection of Hammond et al. [24] to preserve differentiability during binarization.

We obtain the simulated permittivity from the projected design variables through

(7) $$\varepsilon \left(\theta_{\text{sim}}\right)=\varepsilon_{\min}+\theta_{\text{sim}}\left(\varepsilon_{\max}-\varepsilon_{\min}\right),\;\varepsilon_{\min}={\left(n_{\text{eff}}^{150\;nm}\right)}^{2},\varepsilon_{\max}={\left(n_{\text{eff}}^{220\;nm}\right)}^{2}.$$

The modeled cross‐section contains a 150‐nm residual silicon slab, a patterned 70‐nm cap, fixed 220 nm silicon access structures, and silica cladding. Variational mode reduction at λ=1.55μm gives $n_{\text{eff}}^{150\;nm}=2.437$ and $n_{\text{eff}}^{220\;nm}=2.849$ for the stored responses. Electromagnetic simulations use this two‐dimensional effective‐index model and a single quasi‐TE access‐port mode. For input port s, the forward incident amplitude $a_{s}$ is measured at the input reference plane, and the normalized responses are

(8) $$T_{o,s}=\frac{A_{\text{out},o,s}^{+}}{a_{s}},\;R_{i,s}=\frac{A_{\text{in},i,s}^{-}}{a_{s}}.$$

This column‐wise normalization removes the source‐spectrum amplitude and input reference phase.

Let

$$T\left(\theta_{\text{sim}}\right)\in C^{N_{\text{out}}\times N_{\text{in}}},\;R\left(\theta_{\text{sim}}\right)\in C^{N_{\text{out}}\times N_{\text{in}}}$$

denote the transmission and reflection blocks extracted from simulation. We then define the realization objective as

(9) $$L_{\text{realize}}\left(\theta_{\text{raw}}\right)=w_{T}\|T\left(\theta_{\text{sim}}\right)-T^{⋆}{\|}_{F}+w_{R}\|R\left(\theta_{\text{sim}}\right){\|}_{F}+\sum_{j}\mu_{j}\;P_{j}\left(\theta_{\text{sim}}\right).$$

The objective matches the desired local transmission and suppresses reflection. Task metrics are evaluated from the extracted response.

The transmission and reflection terms use unsquared, unnormalized complex Frobenius norms. For input s, the insertion loss is

$${IL}_{s}=-10\log_{10}\left(\sum_{o}|T_{o,s}{|}^{2}\right).$$

We report the arithmetic mean of ${IL}_{s}$ and the largest ${IL}_{s}$ across input ports as the mean and worst‐input insertion losses.

By comparison, a direct geometry‐to‐task formulation would optimize

(10) $$L_{\text{task}}\left(\theta_{\text{raw}};B\right)=\frac{1}{\left|B\right|}\sum_{b\in B}\ell \left(\Psi \left(G_{\text{eval}}\left(\theta_{\text{sim}}\right)a_{b}\right),y_{b}\right),$$

where B is the current minibatch and $G_{\text{eval}}\left(\theta_{\text{sim}}\right)$ denotes the full optical forward map evaluated at the projected design. Under that formulation, each adjoint iteration depends on the sampled data. Once $T^{⋆}$ is fixed, the adjoint sources in the two‐stage workflow depend on the scattering residuals $T\left(\theta_{\text{sim}}\right)-T^{⋆}$ and $R\left(\theta_{\text{sim}}\right)$. The inverse‐design loop is batch‐free, and its cost is independent of the dataset size.

### 2.3 Electromagnetic Solve Accounting

$N_{fwd}$ denotes the number of input‐port forward source simulations and $N_{bwd}$ the number of output‐port adjoint source simulations in one optimizer update. One update uses $N_{fwd}+N_{bwd}=N_{\text{in}}+N_{\text{out}}$ source solves. Reflection is recorded during the input‐port simulations and requires no additional source batch. MedNIST uses 16+6=22 solve‐equivalents per update and 550 over 25 updates. RSSCN7 uses 16+16=32 per update. Yin–Yang uses 4+6=10 per update and 250 over 25 updates. This accounting is additive and independent of the number of images used to train the fixed target operator.

### 2.4 Task Evaluation

Task metrics are computed only after the realized transmission matrix $T\left(\theta_{\text{sim}}\right)$ has been extracted from simulation. For an all‐optical setting,

$$u_{\text{eval}}=T\left(\theta_{\text{sim}}\right)a,\;I_{\text{eval}}=|u_{\text{eval}}{|}^{2},\;g_{\text{eval}}=I_{\text{eval}}.$$

With a fixed downstream evanescent stage, whose forward matrix is E (i.e., O=E), and using the same trained linear readout (W,b), we instead have

$$u_{\text{eval}}=E\;T\left(\theta_{\text{sim}}\right)\;a,\;I_{\text{eval}}=|u_{\text{eval}}{|}^{2},\;g_{\text{eval}}=W^{⊤}I_{\text{eval}}+b.$$

The reported accuracy and cross‐entropy are computed from the logits $g_{\text{eval}}$.

### 2.5 Preprocessing, Encoding, and Locality

To satisfy the available port budget, we preprocess the MedNIST [27] and RSSCN7 [28] datasets before optical encoding. MedNIST contains 58,954 grayscale 64×64 images in six classes: AbdomenCT, BreastMRI, CXR, ChestCT, Hand, and HeadCT. A deterministic stratified split with Seed 7 assigns 48,000 images to training and 10,954 to testing. We flatten and normalize the images to [0, 1], then reduce them to 16 dimensions by principal component analysis [31],

$$x=U_{\text{PCA},r}^{⊤}\left(z-\mu \right),$$

so that the optical input dimension matches the available number of ports. RSSCN7 contains 400 images in each of seven classes. We use a deterministic stratified 80/20 partition, giving 2240 training and 560 test images. PCA and coordinate scaling are fitted only on the training partition for both datasets.

The study uses two optical encoding families

$$\phi_{\text{amp}}\left(x\right)=x,\;\phi_{\text{phase}}\left(x\right)=e^{-i\pi x}.$$

The former is a real‐amplitude encoding, and the latter is a phase‐only encoding on the unit circle. MedNIST uses the real PCA coordinates as amplitudes. For RSSCN7, each training‐fitted PCA coordinate is scaled and clipped to x∈[−1,1], so exp(−iπx) spans [−π,π]. The Yin–Yang task uses a separate amplitude‐phase encoder described below.

A preliminary surrogate‐only sweep over hard half‐bandwidths w=1,…,15 and a dense matrix used 10 predetermined seeds and training‐only validation accuracy. The mean accuracy was 51.90±0.88% at w=5, compared with 50.80±1.23% at w=4, 51.32±1.26% at w=8, and 52.48±0.72% for the dense matrix. Because a hard w=5 matrix retains 146 of 256 entries, we selected w=5 as an accuracy–locality compromise; the full sweep is reported in Supporting Information S1: Figure S2. The reported RSSCN7 router then uses this width as the neighborhood of a differentiable locality constraint rather than as a hard mask. Every complex entry remains trainable, whereas the surrogate loss assigns a 10% power budget to entries outside |i−j|≤5. The selected target is fully dense, places 89.97% of its power within five port spacings, and has a measured out‐of‐band power fraction of 10.03%. The fixed evanescent stage redistributes this locally concentrated router response across the output ports. The reported end‐to‐end operator is the product $ET_{R}$, as illustrated in Figure 2.

**FIGURE 2:**
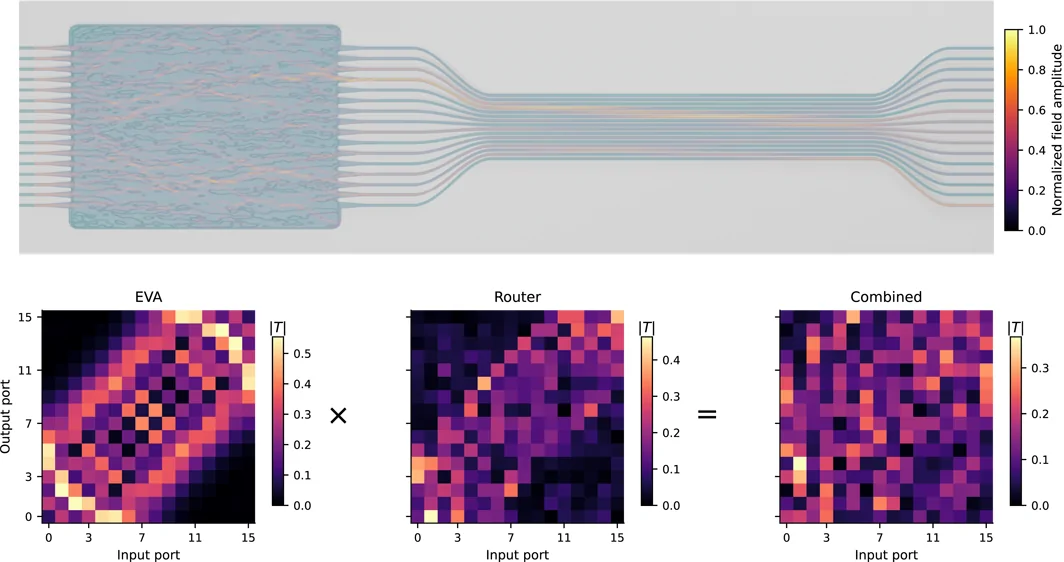
Local router combined with an evanescent array. (Top) Three‐dimensional view of the uniform‐initialized router at optimizer Update 15 and the fixed EVA. The field for RSSCN7 test Sample 0 is colocated with the solver permittivity grids, normalized over silicon pixels, and displayed on the silicon surface. (Bottom) Magnitudes of the EVA response (left), simulated router response (center), and composed response (right), ordered as $ET_{R}^{\left(15\right)}$.

The three datasets studied here instantiate the same framework in different regimes. MedNIST is an all‐optical example with 16 optical inputs and 6 output classes. RSSCN7 uses 16 optical inputs, a learned 16×16 local router, a fixed 16×16 evanescent array (EVA), and a linear readout on detected intensities. The fixed‐stage response is obtained by FDTD. For the Yin–Yang dataset [29], a seed‐7 rejection sampler generates 6000 balanced points inside the unit‐diameter circle. Each accepted point (x,y) is represented by (x,y,1−x,1−y) and assigned to yin, yang, or dot using the two 0.1‐radius dots and the Yin–Yang regions defined by Kriener et al. A deterministic stratified 80/20 split gives 4800 training and 1200 test samples. Each coordinate v∈[0,1] is encoded as $\sqrt{v}\exp\left(-i2\pi v\right)$. The four‐input, six‐output optical structure remains linear in the complex field; the input‐dependent encoding, square‐law detection, and digital readout produce the nonlinear decision boundary in the original coordinates.

## 3 Results

We evaluate the method on three tasks. MedNIST is an all‐optical classification problem in which the realized operator maps a 16‐dimensional feature vector directly to six class channels. RSSCN7 uses a learned 16×16 local router followed by a fixed 16×16 evanescent array and a digital readout. The Yin–Yang dataset tests the same realization procedure on a nonlinear decision boundary produced by coherent encoding, intensity detection, and a digital readout. Together, these tasks probe transfer of a high‐performing surrogate, locality‐constrained routing with fixed downstream mixing, and nonlinear classification. The surrogate comparisons use fixed protocols and report the final surrogate epoch without test‐set model selection. Chance accuracies are 16.67%, 14.29%, and 33.33%, respectively.

### 3.1 All‐Optical MedNIST Classifier

The MedNIST surrogate identifies a 6×16 passive target matrix with 98.38% test accuracy. After 25 realization updates, the simulated device reaches 98.34% test accuracy. At this stage, the relative transmission residual is 0.1278 and the reflection Frobenius norm is 0.0576. Mean input insertion loss is 5.81 dB for the target and 6.72 dB for the realized response, a 0.91 dB difference under the definition above. Figure 3a shows the geometry and field after 25 updates. Panel (b) compares operator matching with direct task‐loss realization under the same initialization and 25‐update budget. Panels (c–e) report the designated update‐25 operator‐matching checkpoint.

**FIGURE 3:**
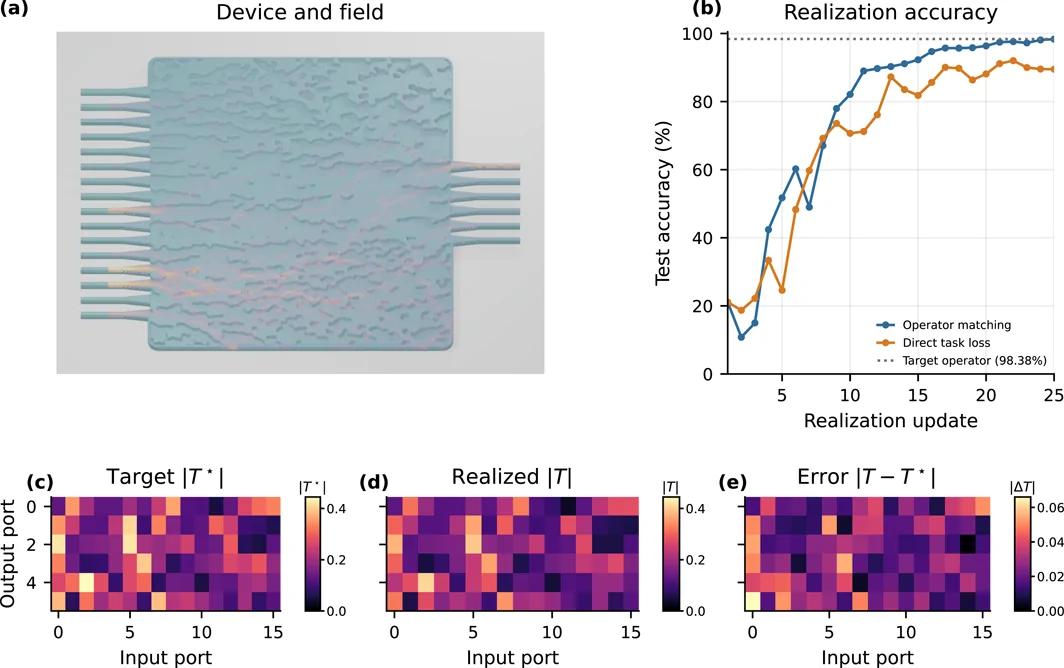
Inverse‐designed MedNIST classifier. (a) Electric‐field amplitude at 1550 nm for a representative HeadCT input after 25 realization updates. The field is normalized over silicon pixels and displayed on the silicon surface. The full simulation domain includes the input and output port planes. (b) Test accuracy during matched physical realizations optimized by complex operator matching and direct task loss; the dotted line marks the 98.38% target‐operator accuracy. (c, d) Target and update‐25 realized transmission magnitudes on a common dimensionless scale. (e) Absolute complex transmission error. The operator‐matched response reaches 98.34% test accuracy.

The matched MedNIST comparison shown in Figure 4 uses 22 solve‐equivalents per update in both branches and 550 solve‐equivalents over 25 updates. Operator matching reaches 98.34% test accuracy, compared with 89.50% for direct normalized‐power cross‐entropy. Elapsed times are 25.5 and 24.7 min, respectively. Operator matching therefore reaches higher final accuracy under this matched budget. The direct task objective permits any transmission matrix that lowers classification loss after photodetection. In this run, it selects an operator whose transmission error relative to $T^{⋆}$ remains nearly constant while its classification loss decreases. Operator matching reduces the transmission error and preserves the target amplitude and phase relations needed for composition with downstream optical stages. Both branches reduce reflection. Figure 5c reports the corresponding three‐initialization comparison for Yin–Yang.

**FIGURE 4:**
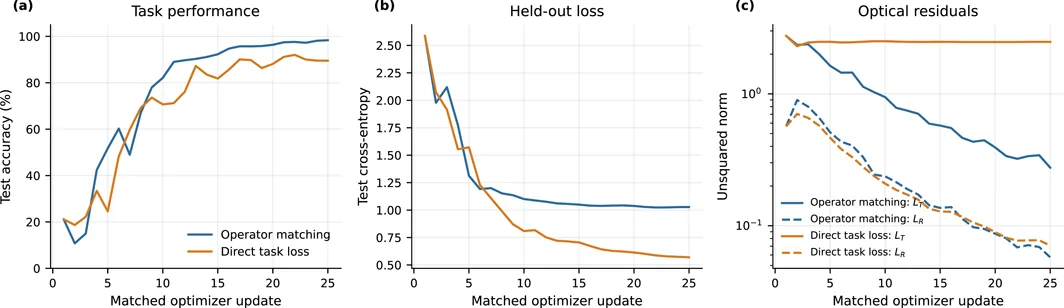
Matched operator and task objectives. Both MedNIST branches start from the same physical density and use the same geometry, fabrication map, optimizer, solver, and 25‐update budget. The panels show (a) test accuracy, (b) test cross‐entropy, and (c) transmission and reflection errors for both objectives. Operator matching progressively reproduces the prescribed complex operator $T^{⋆}$. Direct task optimization instead selects a different operator that improves classification after photodetection without reproducing $T^{⋆}$. Both objectives reduce reflection. Operator matching reaches 98.34% test accuracy in 25.5 min, whereas direct normalized‐power cross‐entropy reaches 89.50% in 24.7 min.

**FIGURE 5:**
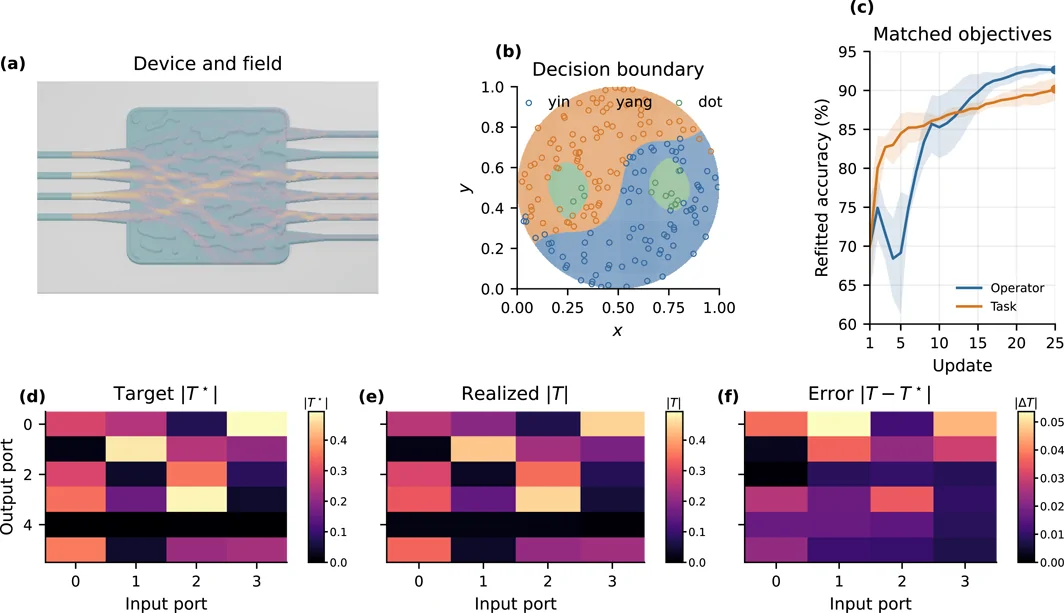
Yin–Yang nonlinear task. (a) Electric‐field amplitude at 1550 nm of the four‐input, six‐output device after optimization. The full simulation domain includes all input and output port planes. (b) Seed‐23 decision boundary obtained with the fixed downstream readout, reaching 93.00% test accuracy. (c) Refitted‐readout accuracy over all 25 updates for matched uniform, seed‐17, and seed‐23 physical initializations. Lines and shaded bands report the mean and sample standard deviation across the three paired runs. At Update 25, the means are 92.64% for operator matching and 90.17% for direct task loss. (d, e) Seed‐23 target and realized 6×4 transmission magnitudes on a common dimensionless scale. (f) Absolute complex transmission error.

### 3.2 Banded Router on RSSCN7

The nominal fixed stage contains 16, 500 nm‐wide silicon strip waveguides with 200 nm edge‐to‐edge gaps and a 47.5 μm coupling section. Ten‐micrometer cosine S‐bends connect the compact coupling pitch to 1.75 μm external pitch, followed by 3.1 μm access sections. The fixed‐stage response in Figure 6 is obtained by FDTD.

**FIGURE 6:**
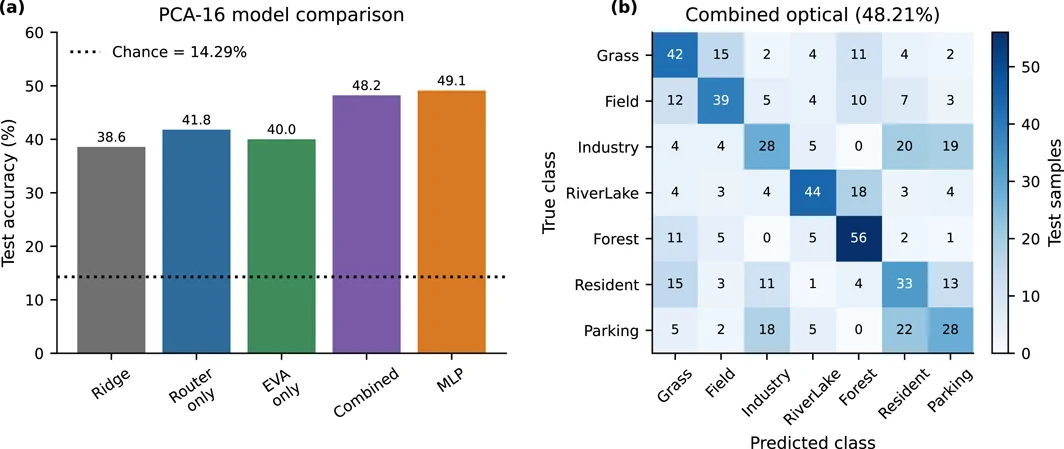
RSSCN7 model comparison. (a) Test accuracy for the ridge classifier, router‐only model, EVA‐only model, combined optical model, and PCA‐16 multilayer perceptron. (b) Test‐set confusion matrix for the combined optical model.

Using the same train‐only PCA‐16 representation, the ridge classifier, router‐only model, EVA‐only model, combined optical model, and PCA‐16 multilayer perceptron obtain 38.57%, 41.79%, 40.00%, 48.21%, and 49.11% test accuracy, respectively. The combined optical model consists of the learned router and readout together with the evanescently coupled waveguide array.

The combined optical model is 0.90% points below the PCA‐16 multilayer perceptron. Both models have their lowest classwise F1 scores on Industry, Resident, and Parking: 0.378, 0.386, and 0.373 for the optical model, compared with 0.387, 0.352, and 0.474 for the multilayer perceptron. The shared Industry and Resident errors identify ambiguity in the common PCA‐16 representation, whereas the additional Parking difference measures a remaining architecture and readout contribution.

The physical target is a fully dense banded router with a 10% out‐of‐band power budget. It places 89.97% of its power within five port spacings and has a measured out‐of‐band power fraction of 10.03%. Its test accuracy before electromagnetic realization is 51.61%.

We also compared realization of the banded router followed by a full‐wave fixed stage with the direct realization of their dense product. This matched control uses the stored FDTD response of the 200 nm‐gap and 47.5 μm fixed stage. Both branches used the same seed‐23 physical initialization, solver, optimizer, fabrication schedule, 15‐update comparison, and dense end‐to‐end target. The factorized branch had lower end‐to‐end transmission residual in 13 of 15 updates. At Update 15, its raw Frobenius residual was 1.3614, compared with 1.5127 for direct realization. Normalizing by the dense‐target Frobenius norm gives 38.13% and 42.37%, respectively; the corresponding reflection norms are 0.0816 and 0.0898. We fitted a multinomial logistic readout to the training‐set optical powers from each update‐15 response and used the test partition only for reporting. The resulting update‐15 accuracies are 50.18% for the factorized branch and 52.14% for the direct branch. Supporting Information S1: Figure S3 shows the projected permittivity, complete residual trajectories, refitted‐readout accuracy trajectories, and end‐to‐end error maps.

### 3.3 Nonlinear Yin–Yang Task

The Yin–Yang experiment uses a four‐input, six‐output freeform device followed by intensity detection and a digital readout. Three 25‐update realizations start from one uniform density and two random freeform initializations (Seeds 17 and 23). Final fixed‐readout accuracies are 89.17%, 92.17%, and 93.00%, giving 91.44±2.02% and a range of 89.17%–93.00%. The transmission residual is 0.110±0.057, the reflection residual is 0.0147±0.0051, and mean input insertion loss is 4.87±0.40 dB.

Each operator‐matching realization is paired with a direct task‐loss realization from the identical physical initialization. A multinomial readout is fitted separately to the training‐set optical powers of each update‐25 response and evaluated on the held‐out test partition. Operator matching reaches 93.17%, 92.42%, and 92.33% for the uniform, seed‐17, and seed‐23 initializations, compared with 88.50%, 91.25%, and 90.75% for direct task loss. The corresponding means are 92.64% and 90.17%, a difference of 2.47% points, with operator matching higher in all three pairs.

### 3.4 Physical Validation

At 1550 nm, the binary Yin–Yang device obtains 92.58% in the 2D effective‐index model and 81.58% in 3D after response‐specific readout refitting. After fitting output phases and one passive amplitude scale, the 2D‐to‐3D transmission‐matrix difference is 0.453. This difference indicates that the compact device relies on strong local scattering. We expect the discrepancy to decrease for a larger design area, where the transformation can be distributed over a longer propagation distance with weaker local scattering. This expectation is particularly relevant because our device occupies a 10.5 μm square, making it more compact than the 35.4 μm × 29.4 μm region used for the 10×10 design of Nikkhah et al. [21]. Its 3D mean and worst‐input insertion losses are 6.31 and 6.84 dB, and normalized reflection is 0.110. The fixed‐readout 2D wavelength sweep gives 90.33%, 90.75%, and 88.00% accuracy at 1540, 1550, and 1560 nm. For Yin–Yang, 20 nm over‐etch and 20 nm under‐etch give 89.08% and 88.00% accuracy, respectively, after readout refitting. For the fabrication‐refined MedNIST checkpoint with a fixed readout, 20 nm over‐etch and 20 nm under‐etch give 59.05% and 65.02%, respectively, compared with 96.57% at the nominal geometry. The 2D‐to‐3D comparison and wavelength sweep use the same binary geometry but different evaluation protocols. The former retrains the electronic classifier for the stored nominal 2D response, whereas the latter independently simulates each wavelength and keeps one classifier fixed across the sweep. This protocol difference gives 92.58% for the nominal 2D comparison and 90.75% for the 1550 nm wavelength‐sweep point.

Input‐phase sensitivity is evaluated from the stored seed‐23 transmission response without additional electromagnetic simulations. Independent Gaussian phase errors are applied after coherent encoding, whereas the readout remains fixed. Across 100 trials, the selected response changes from 93.00% at $\sigma_{\phi }=0{}^{\circ}$ to 91.09±1.38% for a static channel offset and 90.74±0.59% for sample‐dependent jitter at $\sigma_{\phi }=10{}^{\circ}$. At 20°, the corresponding means are 87.43±3.35% and 86.66±0.75%.

## 4 Discussion

The two‐stage workflow separates task learning from full‐wave electromagnetic realization. In the matched MedNIST comparison, operator matching gives higher accuracy under the tested 25‐update budget. Both objectives use 550 solve‐equivalents, and their elapsed times are 25.5 and 24.7 min. Complex operator supervision changes the optimization objective while preserving the electromagnetic work per update.

The Frobenius residual specifies the required amplitude and phase at each output port, so the operator‐matching adjoint sources depend on modal scattering quantities. The direct‐task adjoint sources depend on the detected powers and labels in the predetermined 250‐sample batch. Operator matching reaches higher final accuracy in the MedNIST comparison and all three paired Yin–Yang comparisons under common readout refitting.

The existing controls narrow the physical origin of the excess insertion loss. The RSSCN7 power‐concentration term is applied while learning the target and is therefore already contained in $T^{⋆}$. It does not delete long‐range couplings: every router entry remains trainable, and 10.03% of the selected target power lies outside five port spacings. The target constraint therefore changes the desired power distribution but cannot by itself explain attenuation introduced when the fixed target is transferred to a device. MedNIST provides an independent check because its realized response has 0.91 dB more mean insertion loss than its target even though that architecture has neither a banded target nor the RSSCN7 local‐router/fixed‐mixer factorization. In the matched RSSCN7 control, the factorized branch also has a lower update‐15 end‐to‐end residual than dense direct realization (38.13% vs. 42.37%) and a lower reflection norm (0.0816 vs. 0.0898). These comparisons do not support the routing bandwidth or local factorization as the dominant source of the excess loss.

The response‐level evidence instead points to attenuation introduced by the finite, fabrication‐constrained electromagnetic realization. For RSSCN7, fitting detector‐equivalent output phases and one passive amplitude reduces the relative residual from 0.449 to 0.227. The fitted amplitude of 0.633 corresponds to 40.1% power scaling relative to the target. The matched factorized response has a reflection norm of 0.0816, only 6.0% of its raw transmission‐mismatch norm of 1.3614, so the reported objective discrepancy is not dominated by reflection. We therefore attribute the excess loss primarily to attenuation and scattering incurred while implementing the complex target within the finite footprint under the 200‐nm filter and projection, with the remaining aligned residual measuring operator‐shape error. The stored controls do not vary footprint and projection independently, so they cannot assign separate numerical shares to those two coupled constraints. The 3D comparison additionally measures the response change produced by the extruded cross‐section.

The same constraints suggest operator‐level routes to improve the realizability of future targets without placing the physical‐device model inside the task loss. One option is to construct targets with a strict passivity margin, $\|T_{\text{target}}{\|}_{2}\leq \rho <1$, rather than permit the largest singular value to reach unity. Nikkhah et al. use singular‐value analysis to rescale a prescribed transmission matrix when its largest singular value exceeds one and account for the known scalar outside the passive block [16]. This scaling makes the matrix compatible with passive transmission; it does not guarantee implementation by our finite device. Modular construction provides a second option. A target can be parameterized as a product of simpler passive stages, such as the local router and fixed mixer used here. Nikkhah et al. likewise combine a reconfigurable coupler network with an inverse‐designed operator block and provide an auxiliary multimode waveguide for power not directed to the monitored outputs. A larger design region supplies more electromagnetic degrees of freedom, as reported by Camacho et al. [15], but increases footprint. These are prospective target‐construction and architecture choices. The reported realizations retain the fixed task‐trained targets without retrospective scaling, projection, or replacement.

For MedNIST, the target matrix predicts a mean insertion loss of 5.81 dB, whereas the realized device gives 6.72 dB. The device therefore has 0.91 dB of additional loss. Expressed as power, it transmits 21.3% of the input power, compared with 26.2% predicted by the target, or 81.1% of the target transmission. We then tested whether this difference arises mainly from a uniform reduction in power or from distortion of the intended input–output transformation. Before comparing the two matrices, we removed one phase offset from each output and fitted one common amplitude factor. The output‐phase offsets do not affect intensity detection, and a common amplitude factor does not change the predicted class because it scales all output intensities equally. The fitted amplitude is 0.897, corresponding to a power factor of $0.897^{2}=80.5\%$. This value closely matches the independently calculated 81.1% realized‐to‐target power ratio. The relative matrix error also decreases from 0.128 to 0.047 after alignment. These results indicate that most of the MedNIST discrepancy is a nearly uniform loss of optical power; the remaining error represents nonuniform changes in the relative amplitudes and phases of the matrix elements. The same analysis reduces the relative error from 0.449 to 0.227 for RSSCN7 and from 0.041 to 0.028 for Yin–Yang. We nevertheless optimize the complete complex matrix because both amplitude and phase determine the response of subsequent optical stages. Directly rewarding total transmitted power could reduce insertion loss while changing the intended input–output transformation.

Sved et al. [23] also exploit electromagnetic linearity. Each geometry update uses a fixed set of $N_{\text{in}}+N_{\text{out}}$ basis simulations, and superposition reconstructs the training‐sample fields. Their physical optimization remains coupled to the training data: A labeled minibatch of size B=250 supplies the geometry gradient. A complete pass through $N_{\text{data}}$ samples requires $\left\lceil N_{\text{data}}/B\right\rceil$ minibatch updates. The basis‐solve count per update stays fixed, whereas batch size controls the samples and task‐evaluation work represented in each geometry update.

In our workflow, task learning is completed before electromagnetic realization. The physical optimizer matches a fixed complex scattering matrix and therefore uses no samples, labels, minibatches, or dataset passes. Each realization update requires exactly $N_{\text{in}}+N_{\text{out}}$ solve‐equivalents, and the number of physical updates is independent of $N_{\text{data}}$ and B. The comparable six‐class example of Sved et al. uses approximately (15+6)×150=3150 solve‐equivalents. The matched MedNIST comparison here uses (16+6)×25=550 solve‐equivalents for each objective. Operator matching reaches higher final accuracy at the matched internal budget and uses fewer physical updates than the published comparison.

The sensitivity studies separate variability in the matrix‐surrogate and electromagnetic‐realization stages. Eight predetermined RSSCN7 surrogate seeds give 50.87±1.15% test accuracy at the fixed final epoch, with a range of 48.75%–52.32%. Three Yin–Yang freeform initializations give 91.44±2.02% fixed‐readout accuracy and a transmission residual of 0.110±0.057. The accuracy is more stable than the realized complex matrix, indicating that different physical local solutions can preserve similar classification performance.

The fixed‐stage study contains 12 FDTD responses spanning three waveguide gaps and four coupling lengths. For each response, the router and electronic readout are trained together using five predetermined seeds. The training‐only validation means range from 54.68% to 55.70%, measuring the coupled response of the fixed stage and learned router across the sampled geometries.

The reported sensitivity estimates apply to a device class with a fixed design‐region size, 25 nm discretization, 200 nm fabrication filter, platform cross section, and locality width w=5. A broader optimization could place these variables in an outer loop using early truncated physical runs, learned geometry representations, and Bayesian optimization. HiLAB combines these elements for nanophotonic inverse design [32], and related representation‐learning strategies are reviewed by Marzban et al. [33].

## 5 Conclusions

We introduced a surrogate‐guided inverse‐design framework for compact photonic neural networks. Task training is performed on a passive complex matrix with bounded singular values, and electromagnetic realization is posed as a fixed‐target adjoint problem driven by complex transmission matching and reflection suppression. This separation fixes the electromagnetic work per update through the input and output port counts and makes it independent of training‐set size. In the matched MedNIST comparison, operator matching reaches 98.34% accuracy, compared with 89.50% for direct task optimization at the same 550‐solve budget. The selected RSSCN7 banded router is fully dense and uses a power‐concentration term centered at w=5, with 10.03% measured out‐of‐band power. In the matched seed‐23 physical comparison through Update 15, the factorized branch has lower end‐to‐end transmission residual in 13 of 15 updates and finishes at 38.13%, compared with 42.37% for dense‐direct realization. The Yin–Yang experiments give 91.44±2.02% across three operator‐matching initializations. Under common readout refitting, the three matched objective pairs give 92.64% mean accuracy for operator matching and 90.17% for direct task loss. The physical evaluations cover 2D and 3D responses, wavelength, uniform over‐etch and under‐etch, and phase noise. These results establish surrogate‐guided operator matching for the tested nanophotonic neural networks.

## Author Contributions

**Azka Maula Iskandar Muda:** investigation, writing – original draft, methodology, validation, visualization, writing – review and editing, data curation, formal analysis. **Uğur Teğin:** conceptualization, investigation, funding acquisition, writing – original draft, methodology, validation, visualization, writing – review and editing, project administration, data curation, formal analysis, supervision.

## Funding

This work was supported by the Optica Challenge Award.

## Ethics Statement

The authors have nothing to report.

## Informed Consent

The authors have nothing to report.

## Conflicts of Interest

The authors declare no conflicts of interest.

## Supporting information


Supporting Information S1

## Data Availability

The tutorial notebook and code are available in a public GitHub repository (https://github.com/amiskandarmuda/surrogate‐guided‐inverse‐designed‐nanophotonic‐neural‐network). A version of the notebook will also be published in the Tidy3D tutorials. The tutorial notebook and code are available in the Tidy3D Community Notebooks (https://www.flexcompute.com/tidy3d/community/notebooks/SurrogateGuidedPhotonicClassifier/). No training data are redistributed. MedNIST is provided under CC BY‐SA 4.0; its source images retain their original copyright and usage terms. RSSCN7 contains Google Earth imagery and does not state a license; reuse remains subject to the rights holders’ terms. The Yin–Yang generator is copyright 2020 Laura Kriener and licensed under MIT.
