# Supplementary material for: Surrogate Scattering Matrix‐Guided Inverse Design of Nanophotonic Neural Networks
Source: Nanophotonics. 2026 Sep 23;15(18):e70302. doi: 10.1002/nap2.70302 (PMC13672145; doi:10.1002/nap2.70302)
Supplement: Supplementary file 1 [file NAP2-15-e70302-s001.pdf]

Supplementary Information: Surrogate Scattering Matrix Guided Inverse Design of Nanophotonic Neural Networks

Azka Maula Iskandar Muda and Uğur Teğin

1 Dataset preparation and evaluation

1.1 MedNIST

The MedNIST dataset is obtained from the public MONAI extra-test-data release 0.8.1. It contains 58,954 grayscale 64×64 images in six classes: AbdomenCT, BreastMRI, CXR, ChestCT, Hand, and HeadCT. A seed-7 deterministic stratified split assigns 48,000 images to training and 10,954 to testing. PCA with 16 components is fitted only to the training images. The optical encoder applies the resulting real coordinates as input amplitudes. Chance accuracy is 16.67%.

1.2 RSSCN7

RSSCN7 contains seven classes with 400 images per class. We use a deterministic stratified 80/20 partition, giving 2,240 training and 560 test images. Images are resized to 64×64 RGB, flattened, and reduced to 16 PCA coordinates. PCA and coordinate scaling are fitted only on the training partition. Clipped coordinates $x \in [-1, 1]$ are encoded as $\exp(-i\pi x)$, spanning the phase interval $[-\pi, \pi]$. The test partition is held out during target and model selection. Chance accuracy is 14.29%.

1.3 Yin–Yang

The seed-7 generator draws points uniformly within the unit square, rejects points outside the unit-diameter circle, and balances the yin, yang, and dot classes during acceptance. It generates 6,000 points and represents each accepted coordinate (x, y) as $(x, y, 1-x, 1-y)$. The class rule uses the two 0.1-radius dots and the Yin–Yang regions defined by Kriener, Göltz, and Petrovici. A deterministic stratified 80/20 split gives 4,800 training and 1,200 test samples. Each coordinate $v \in [0, 1]$ is encoded as $\sqrt{v}\exp(-i2\pi v)$. Chance accuracy is 33.33%.

Table S1: RSSCN7 models using the same PCA-16 input representation.

Model	Test accuracy (%)	Role
Ridge classifier	38.57	Linear baseline
PCA-16 MLP	49.11	Digital reference
Router only	41.79	Optical ablation
EVA only	40.00	Optical ablation
Combined optical model	48.21	Router, readout, and evanescently coupled waveguides

The combined optical model is 0.90 percentage points below the PCA-16 multilayer perceptron. Their classwise F1 scores are respectively 0.378 and 0.387 for Industry, 0.386 and 0.352 for Resident, and 0.373 and 0.474 for Parking. The shared weak Industry and Resident results occur in the common PCA-16 feature space. The larger Parking difference quantifies the additional architecture and readout error for that class.

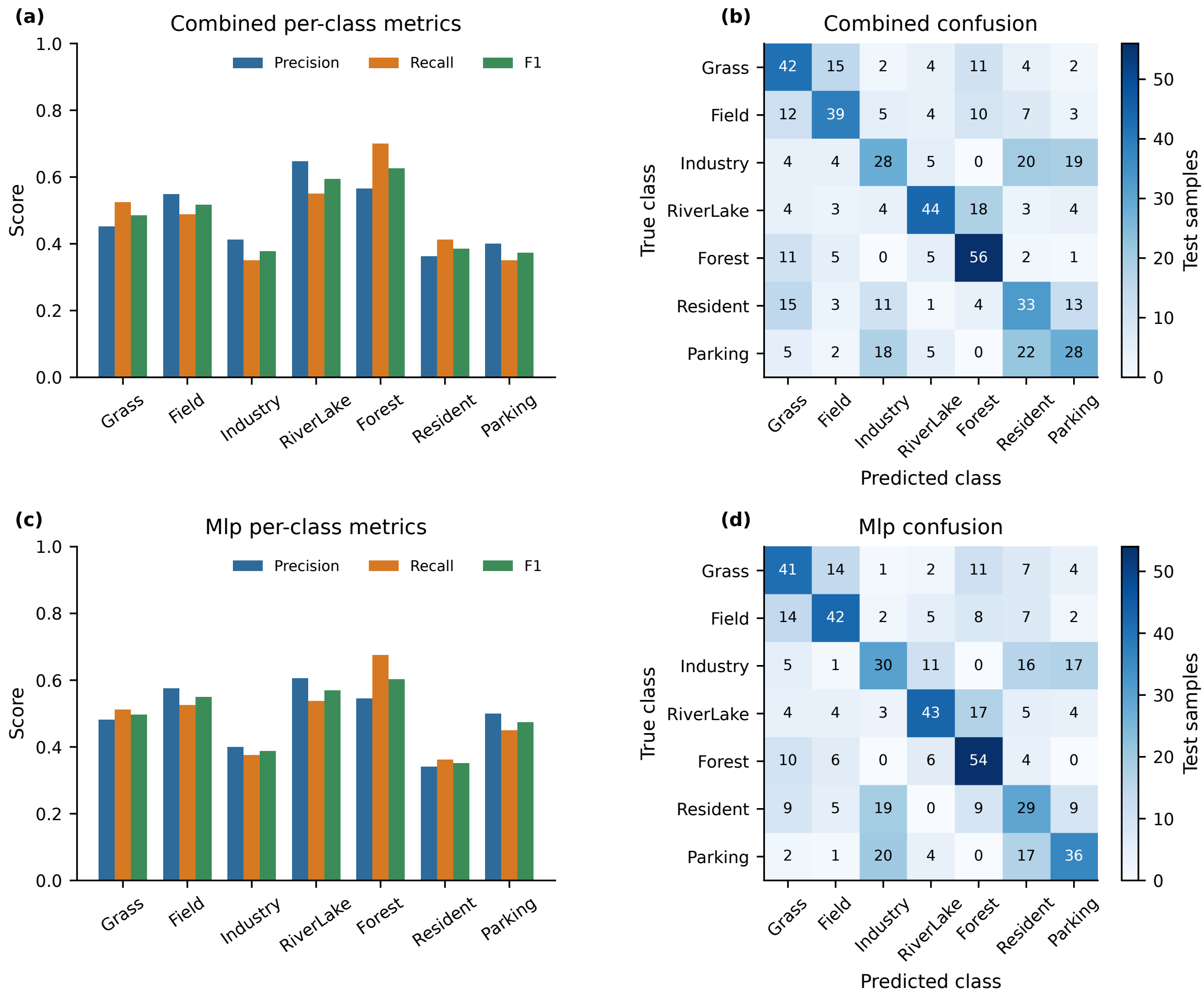

True class \ Predicted class (b) Combined	Grass	Field	Industry	RiverLake	Forest	Resident	Parking
Grass	42	15	2	4	11	4	2
Field	12	39	5	4	10	7	3
Industry	4	4	28	5	0	20	19
RiverLake	4	3	4	44	18	3	4
Forest	11	5	0	5	56	2	1
Resident	15	3	11	1	4	33	13
Parking	5	2	18	5	0	22	28

True class \ Predicted class (d) Mlp	Grass	Field	Industry	RiverLake	Forest	Resident	Parking
Grass	41	14	1	2	11	7	4
Field	14	42	2	5	8	7	2
Industry	5	1	30	11	0	16	17
RiverLake	4	4	3	43	17	5	4
Forest	10	6	0	6	54	4	0
Resident	9	5	19	0	9	29	9
Parking	2	1	20	4	0	17	36

Figure S1: RSSCN7 per-class precision, recall, F1 score, and annotated confusion matrices for the PCA-16 multilayer perceptron and combined optical model.

Table S2: MedNIST per-class metrics for the update-25 realized response.

Class	Precision	Recall	F1 score	Test samples
AbdomenCT	0.9867	0.9968	0.9917	1858
BreastMRI	0.9856	0.9904	0.9880	1664
CXR	0.9714	0.9871	0.9792	1858
ChestCT	0.9810	1.0000	0.9904	1858
Hand	0.9867	0.9586	0.9724	1858
HeadCT	0.9895	0.9682	0.9788	1858

Table S3: Yin–Yang per-class metrics for the seed-23 update-25 response.

Class	Precision	Recall	F1 score	Test samples
Yin	0.9481	0.9325	0.9402	548
Yang	0.9478	0.9170	0.9321	554
Dot	0.7760	0.9898	0.8700	98

2 Passive surrogate parameterization

For an $M \times N$ operator, the surrogate uses

$$T = U\Sigma V^{\dagger}, \tag{1}$$

where U and V are generated from matrix exponentials of skew-Hermitian parameters. A sigmoid bounds each singular value below one. This construction makes T a contraction. For the complete two-sided response

$$S = \begin{pmatrix} R_{\mathrm{L}} & T_{\mathrm{RL}} \\ T_{\mathrm{LR}} & R_{\mathrm{R}} \end{pmatrix}, \tag{2}$$

reciprocity gives $S = S^{\mathsf{T}}$ and therefore $T_{\mathrm{RL}} = T_{\mathrm{LR}}^{\mathsf{T}}$. Passivity gives $S^{\dagger}S \preceq I$; for left incidence, $R_{\mathrm{L}}^{\dagger}R_{\mathrm{L}} + T_{\mathrm{LR}}^{\dagger}T_{\mathrm{LR}} \preceq I$. The contraction parameterization satisfies this necessary transmission condition. Finite freeform extent, port geometry, and the fabrication map further restrict the realizable set. A target outside that set has a positive minimum residual, and a decreasing optimization trace approaches this nonzero plateau.

RSSCN7 jointly trains the optical router and a seven-output affine readout after square-law detection. Training uses the complete 2,240-image training partition at every surrogate update. The reporting protocol uses the fixed final epoch for every seed and condition.

3 Surrogate initialization statistics

The RSSCN7 initialization study uses predetermined seeds 7, 17, 23, 31, 47, 59, 71, and 83. Their fixed-final-epoch test accuracy is $50.87 \pm 1.15\%$, with a range of 48.75–52.32%. These values measure sensitivity of the matrix surrogate.

4 Banded router

We selected the locality scale using a preliminary surrogate-only sweep over hard half-bandwidths $w = 1, \ldots, 15$ and a dense matrix across ten predetermined seeds. Model comparison used training-only validation accuracy. The mean accuracy was $50.80 \pm 1.23\%$ at $w = 4$, $51.90 \pm 0.88\%$ at $w = 5$, $51.32 \pm 1.26\%$ at $w = 8$, and $52.48 \pm 0.72\%$ for the dense matrix. A hard $w = 5$ matrix contains 146 of 256 entries, or 57.0% of the dense parameter count. We therefore selected $w = 5$ as an accuracy–locality compromise. This surrogate sweep determines the locality scale used in the subsequent electromagnetic realization.

The RSSCN7 realization target uses a differentiable penalty that keeps optical power near the corresponding input-port position. Every complex router entry remains trainable. With a five-port neighborhood, the surrogate loss assigns a 10% power budget to entries outside $|i - j| \leq 5$, and the selected

target places 89.97% of its power within five port spacings. Its measured out-of-band power fraction is 10.03%, and its test accuracy before electromagnetic realization is 51.61%.

5 Fixed evanescent array

The nominal stage contains sixteen 500 nm-wide silicon strip waveguides with 200 nm edge-to-edge gaps and a 47.5 µm coupling section. Cosine S-bends of length 10 µm connect the compact coupling pitch to 1.75 µm external pitch, followed by 3.1 µm access sections. The fixed-stage response in Fig. 5 is obtained by FDTD.

A separate geometry study sampled gaps of 100, 150, and 200 nm and coupling lengths of 42.5, 45.0, 47.5, and 50.0 µm, producing twelve stored FDTD stage responses. For each response, the optical router and electronic readout were trained together using five predetermined seeds. Model comparison used training-only validation accuracy, and every seed was reported under the same fixed-epoch rule. The condition means range from 54.68 to 55.70%. This range measures the coupled sensitivity of the learned router and fixed stage to gap and coupling length.

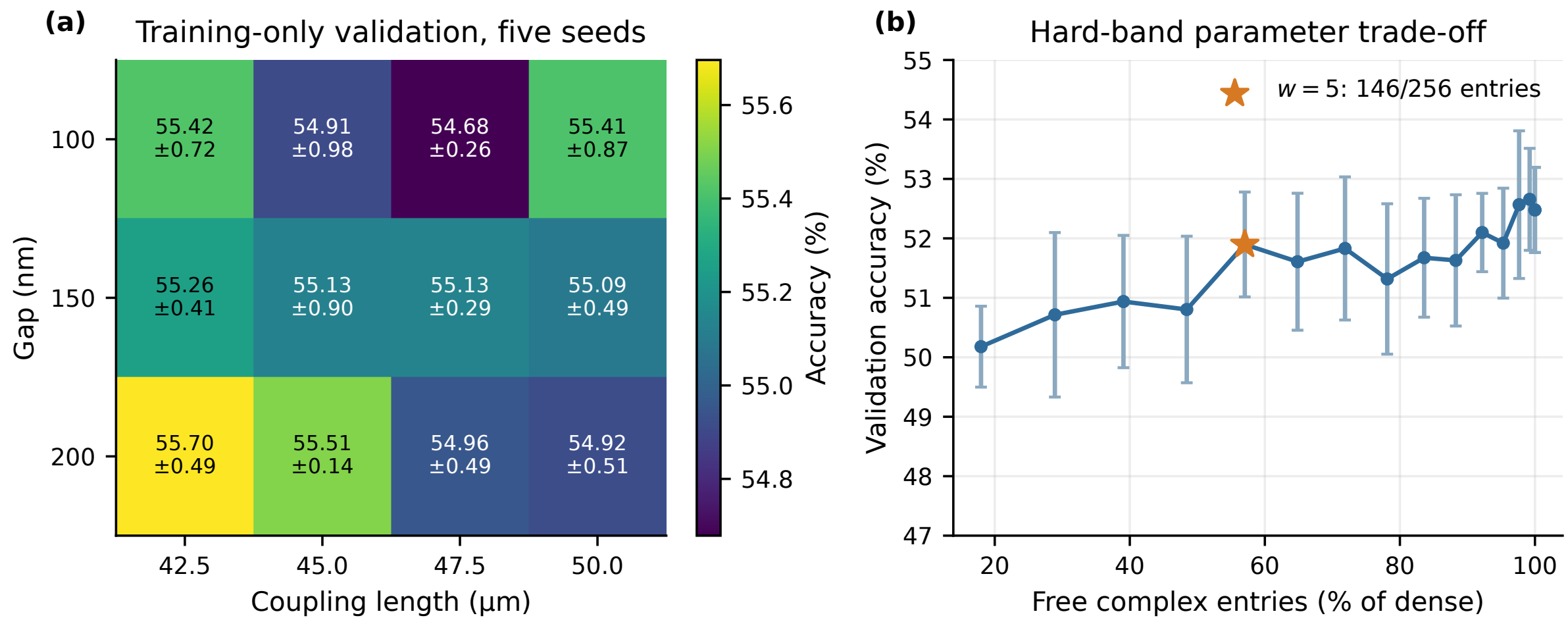

Figure S2: RSSCN7 surrogate selection studies. (a) Five-seed training-only validation accuracy across twelve fixed-stage gap and coupling-length conditions. (b) Training-only validation accuracy across ten predetermined seeds for hard half-bandwidths $w = 1, \dots, 15$ and the dense matrix, plotted against the fraction of retained complex entries. Points and error bars show the mean and sample standard deviation. The selected $w = 5$ retains 146 of 256 entries in the hard-bandwidth sweep.

6 Matched RSSCN7 realization comparison

The matched physical comparison uses the stored full-wave response of the 200 nm-gap, 47.5 µm fixed stage. The factorized branch realizes the banded router and forms its effective transmission as $T_{\mathrm{eff}} = ET_{\mathrm{router}}$, where E is the fixed-stage transmission. The direct branch realizes the dense product $T^{\star}_{\mathrm{dense}} = ET^{\star}_{\mathrm{router}}$ in one freeform region. The matrix identity was verified numerically before comparison.

Both branches use the same seed-23 initial permittivity, square 33.6 µm design region, solver, optimizer, fabrication schedule, and 15-update comparison. The end-to-end transmission loss is $L_T = \|T_{\mathrm{eff}} - T^{\star}_{\mathrm{dense}}\|_F$ for the factorized branch and $L_T = \|T - T^{\star}_{\mathrm{dense}}\|_F$ for the direct branch. The factorized value is lower in 13 of 15 updates.

For every stored response, a multinomial logistic readout is fitted to the training-set optical powers and then evaluated on the test partition. The table reports update 15, and the trajectory shows the response evolution through that update. At update 15, factorization reduces the normalized transmission residual by 4.24 percentage points. The test accuracy after retraining the electronic classifier is 1.96 percentage points lower than the dense-direct branch.

Table S4: Matched RSSCN7 physical realization at update 15. The normalized residual is $100 L_T/\|T^\star_{\mathrm{dense}}\|_F$. Readouts are refitted using only training-set optical powers. The factorized row includes the fixed-stage response.

Realization	L_T	Residual (%)	$\|R\|_F$	Accuracy (%)
Factorized	1.3614	38.13	0.0816	50.18
Dense direct	1.5127	42.37	0.0898	52.14

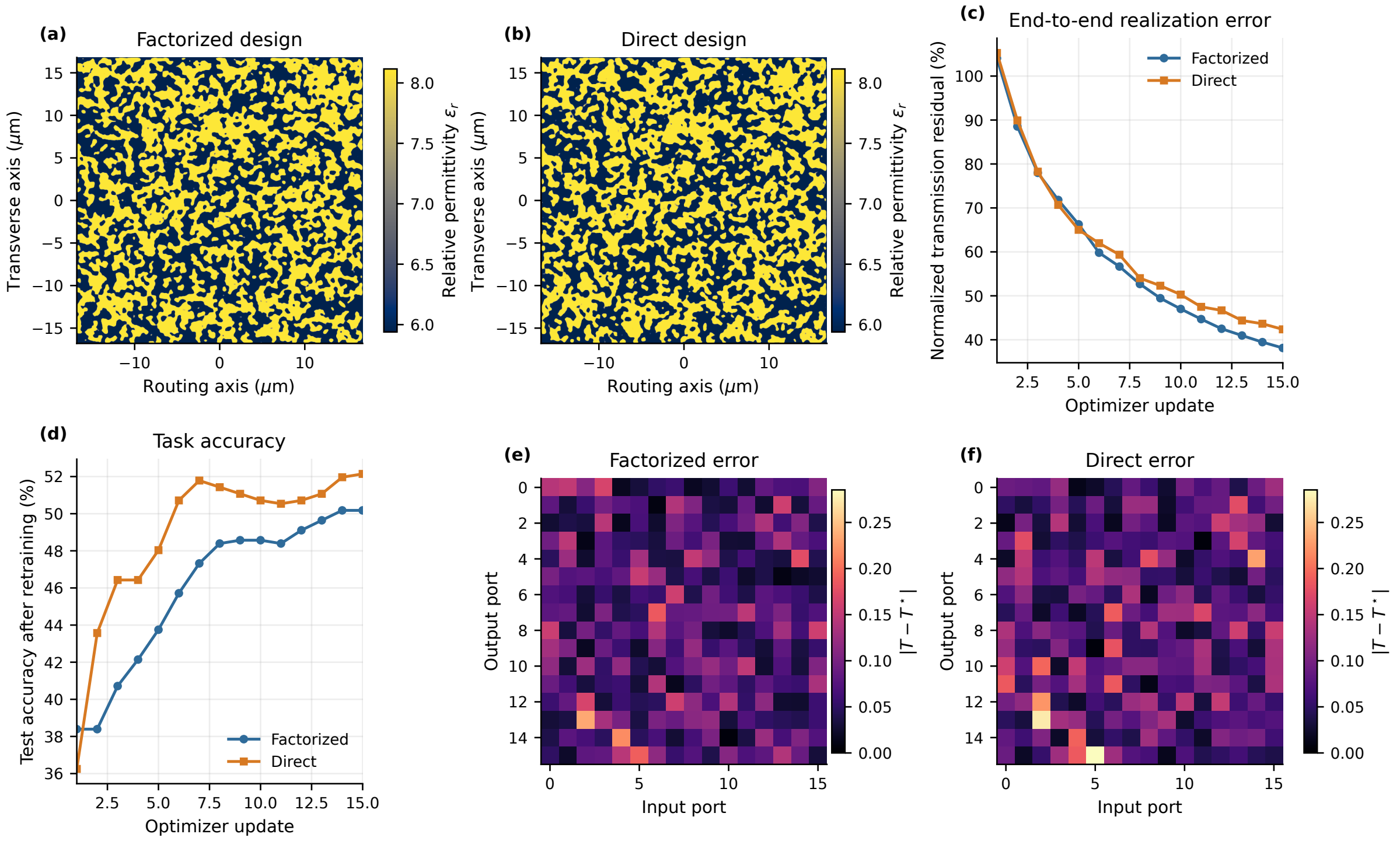

Figure S3: Matched RSSCN7 factorized and dense-direct realizations. (a,b) Update-15 projected relative-permittivity maps. (c) End-to-end normalized complex transmission residual through update 15. (d) Update-15 test accuracy after training a separate multinomial logistic classifier on each response's training-set optical powers. (e,f) Absolute end-to-end complex-amplitude errors at update 15 on a common scale.

7 Matched operator and task objectives

The MedNIST comparison starts both branches from the same physical density and uses the same geometry, fabrication map, optimizer, solver, and 25-update budget. The task branch uses one predetermined batch of 250 samples per geometry update and normalized optical-power cross-entropy. The operator branch uses the complex transmission target and reflection loss. Full-training and full-test metrics are evaluated after each optimizer-forward evaluation. The task gradient uses the predetermined 250-sample batch.

Table S5: Matched MedNIST comparison after 25 updates.

Objective	Test accuracy (%)	Time (min)	Solves/update	Total solves
Operator matching	98.34	25.5	22	550
Direct task loss	89.50	24.7	22	550

Operator matching reaches higher accuracy under the matched electromagnetic budget.

After retraining the electronic classifier separately for each response, operator matching is higher in all three initialization pairs. The mean difference is 2.47 percentage points.

Table S6: Matched Yin–Yang comparison after 25 updates. Each objective pair starts from an identical physical parameter array. For each stored response, the electronic classifier is retrained using only its training-set optical powers.

Initialization	Fixed readout (%)		Classifier retrained (%)	
	Operator	Task	Operator	Task
Uniform	89.17	89.58	93.17	88.50
Seed 17	92.17	89.83	92.42	91.25
Seed 23	93.00	90.58	92.33	90.75
Mean	91.44	90.00	92.64	90.17

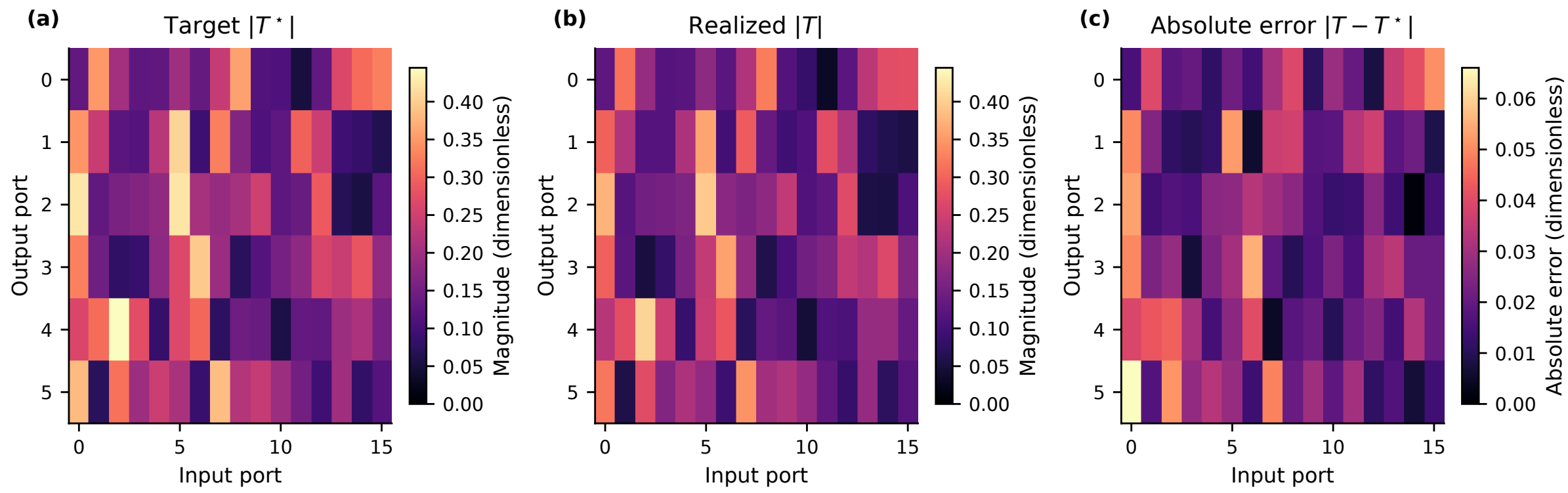

Figure S4: MedNIST update-25 operator response. (a,b) Target and realized transmission magnitudes on a common dimensionless scale. (c) Absolute complex-amplitude error.

8 Separating phase and loss from matrix-shape error

The target and realized transmission matrices can differ in ways that do not change the predicted class. A phase shift applied to an entire output row does not change the detected intensity at that output, and multiplying every output by the same positive amplitude does not change which output is largest. We therefore report both the direct matrix error and the error that remains after fitting these two effects. The fitted error is

$$\min_{D,\ 0\leq\alpha\leq 1} \frac{\|DT_{\text{realized}} - \alpha T^\star\|_F}{\|T^\star\|_F}, \tag{3}$$

where $T^\star$ is the trained target matrix, T_{realized} is the simulated physical-device matrix, D applies one fitted phase shift to each output row, α is one fitted amplitude scale for the full matrix, and $\|\cdot\|_F$ is the Frobenius norm. The fitted phases and amplitude separate detector-equivalent phase offsets and uniform attenuation from the remaining matrix-shape error. The reported classification results use the simulated device response.

Table S7: Relative transmission-matrix error before and after fitting output-row phases and one global amplitude scale.

Task	Direct error	Error after fit	Fitted amplitude
MedNIST	0.128	0.047	0.897
RSSCN7	0.449	0.227	0.633
Yin–Yang	0.041	0.028	0.972

For MedNIST, the direct matrix error is 0.128 and decreases to 0.047 after the fit. The target and realized responses transmit 26.2% and 21.3% of the input power on average, respectively, giving a ratio of 81.1%. The fitted amplitude is 0.897, equivalent to 80.5% in power. The agreement between 81.1% and 80.5% indicates that a nearly uniform loss accounts for most of the insertion-loss difference. The remaining error of 0.047 measures differences in the relative complex coupling between input and output ports.

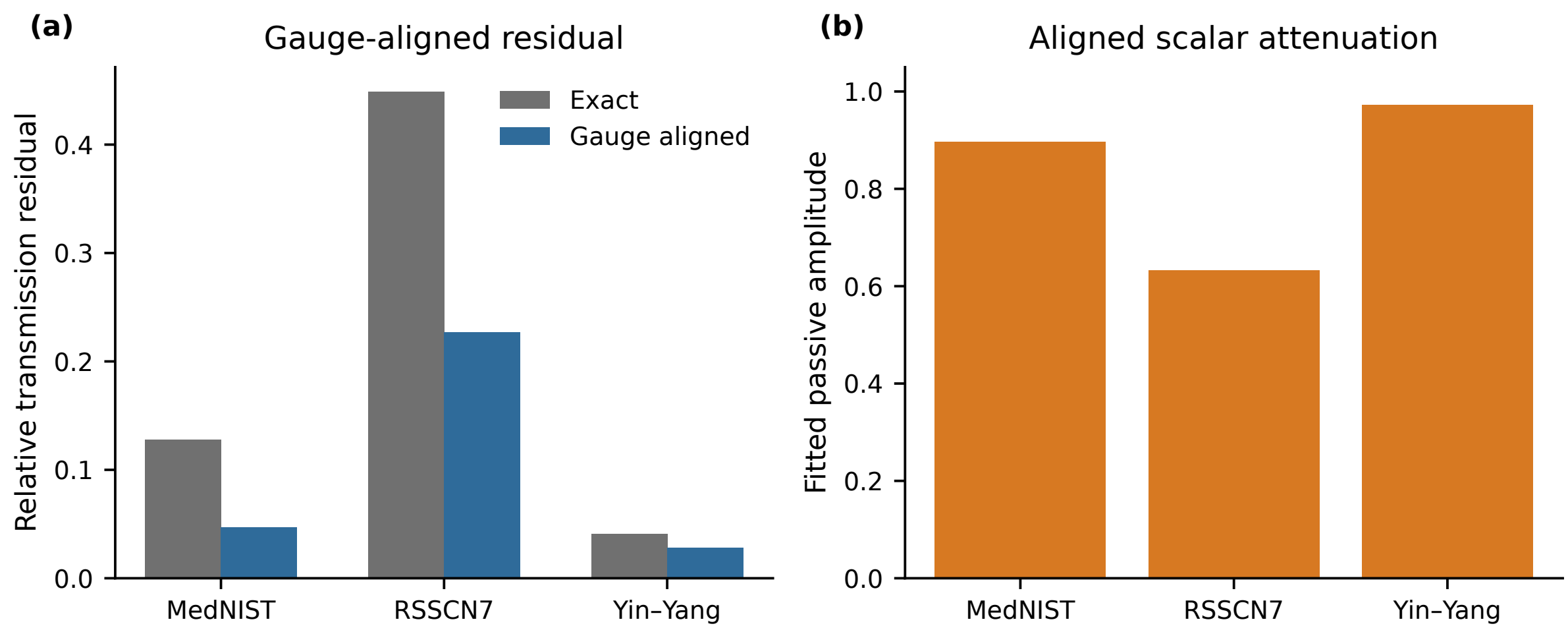

Figure S5: Relative transmission-matrix error before and after fitting output-row phases and one global amplitude scale.

9 Physical-initialization sensitivity

Three 25-update Yin–Yang realizations use one uniform density and two random freeform initializations (seeds 17 and 23). Final accuracies are 89.17%, 92.17%, and 93.00%, giving $91.44 \pm 2.02\%$ and a range of 89.17–93.00%. The transmission residual is 0.110 ± 0.057, the reflection residual is 0.0147 ± 0.0051, and mean input insertion loss is 4.87 ± 0.40 dB.

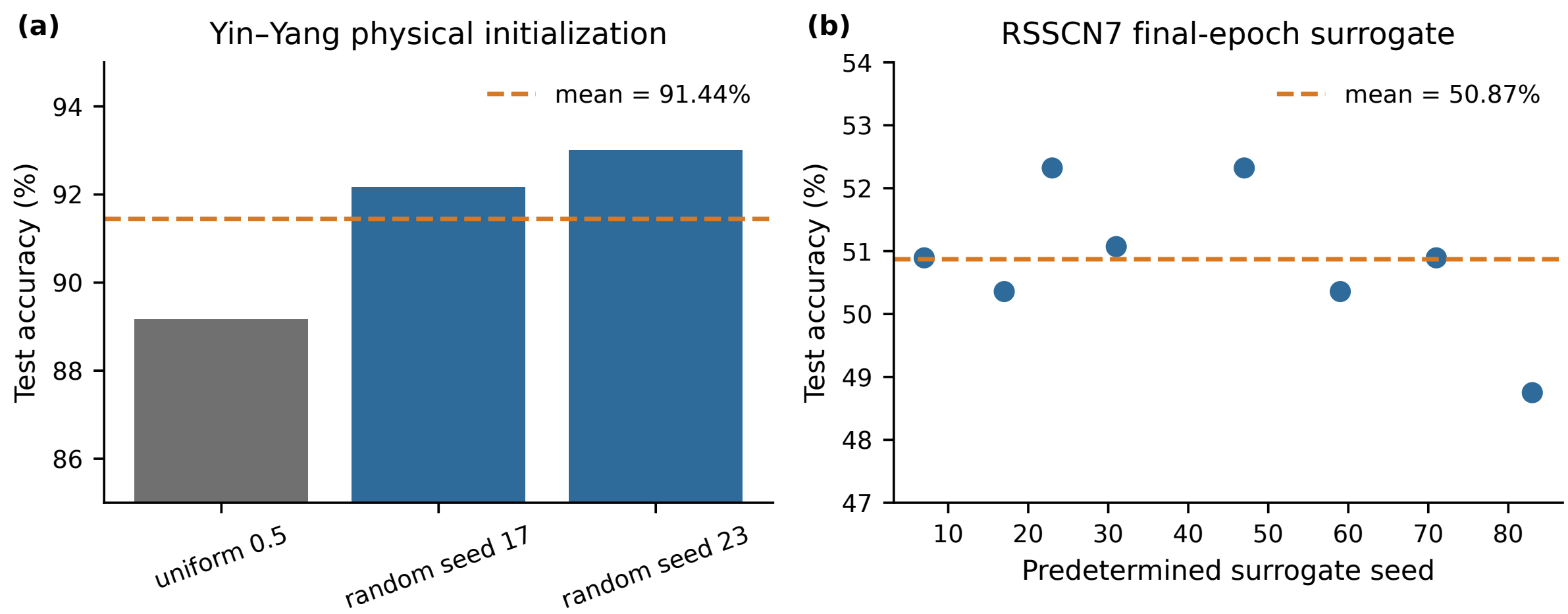

Figure S6: Initialization sensitivity. (a) Three 25-update Yin–Yang freeform realizations. (b) Eight predetermined RSSCN7 surrogate seeds reported at the fixed final epoch.

10 Physical cross section

The modeled cross section contains a 150 nm residual silicon slab, a patterned 70 nm cap, fixed 220 nm silicon access structures, and silica cladding. Variational mode reduction at 1550 nm gives effective indices 2.437 and 2.849 for the 150 and 220 nm states, respectively. The input excitations use the single quasi-TE access-port mode.

11 Dimensionality, spectral, fabrication, and encoding response

A nominal-wavelength full-device Yin–Yang comparison gives 92.58% accuracy in the 2D effective-index model and 81.58% in 3D after retraining the electronic classifier separately for each optical response. After fitting output phases and one passive amplitude scale, the 2D-to-3D transmission-matrix difference is 0.453. This difference indicates that the compact device relies on strong local scattering. Increasing the design area can distribute the transformation over a longer propagation distance, reduce the required local scattering strength, and decrease the resulting 2D-to-3D discrepancy. The 3D mean and worst-input insertion losses are 6.31 and 6.84 dB. The reflection amplitude per input, $\|R\|_F/\sqrt{N_{\text{in}}}$, is 0.110.

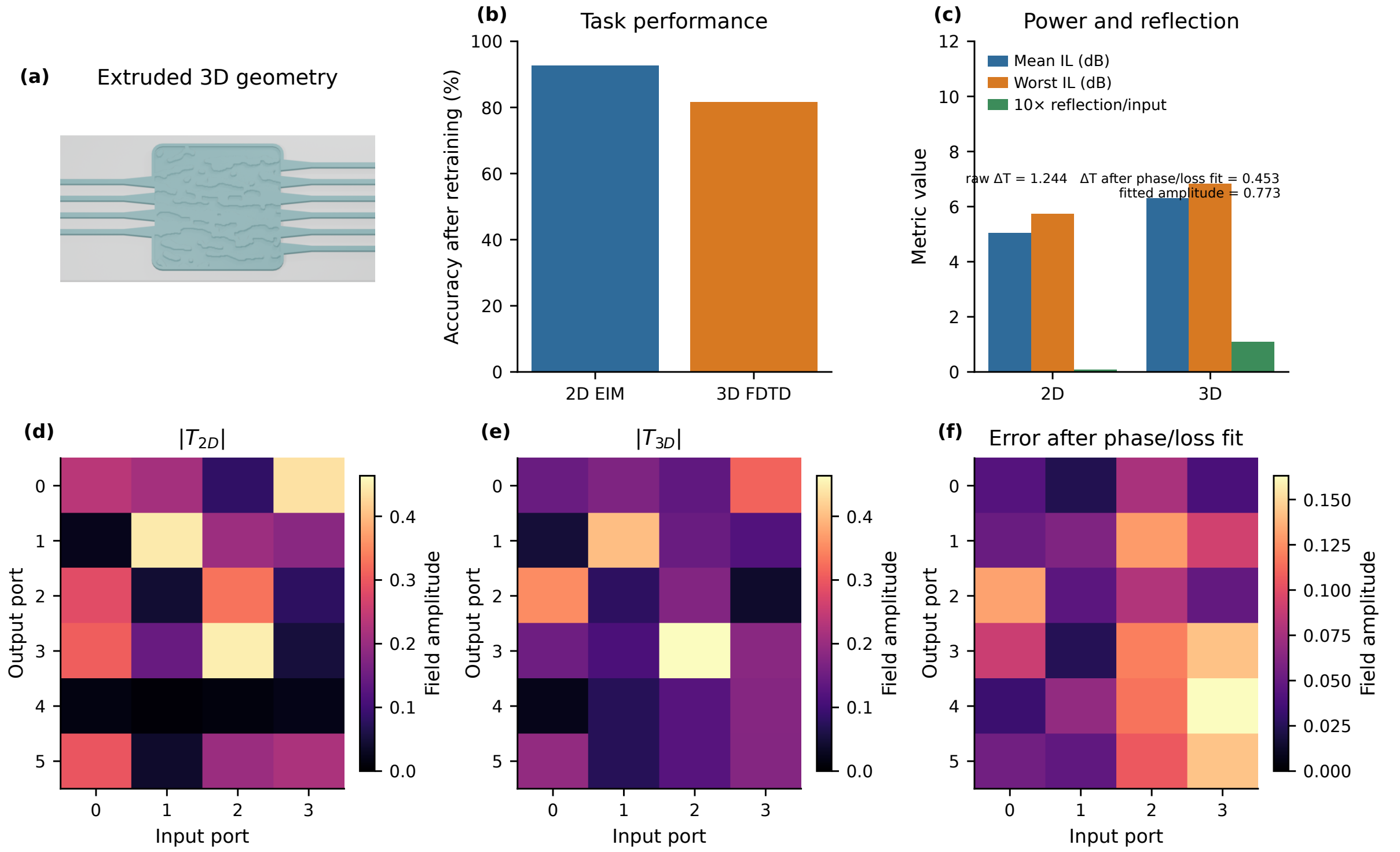

Figure S7: Full-device 2D-to-3D validation of the robust binary Yin–Yang geometry at 1550 nm. (a) Extruded geometry used for the comparison. (b,c) Accuracy after retraining the electronic classifier, insertion loss, and reflection. (d–f) Magnitudes of the 2D response, 3D response, and 3D–2D matrix error after fitting output phases and one amplitude scale. The electronic classifier is retrained separately for the 2D and 3D responses.

The independent 2D wavelength sweep keeps the trained readout fixed. Accuracy is 90.75% at 1550 nm and ranges from 76.42% to 90.33% over the off-nominal 1510–1590 nm points. The mean input insertion loss ranges from 4.95 to 5.42 dB.

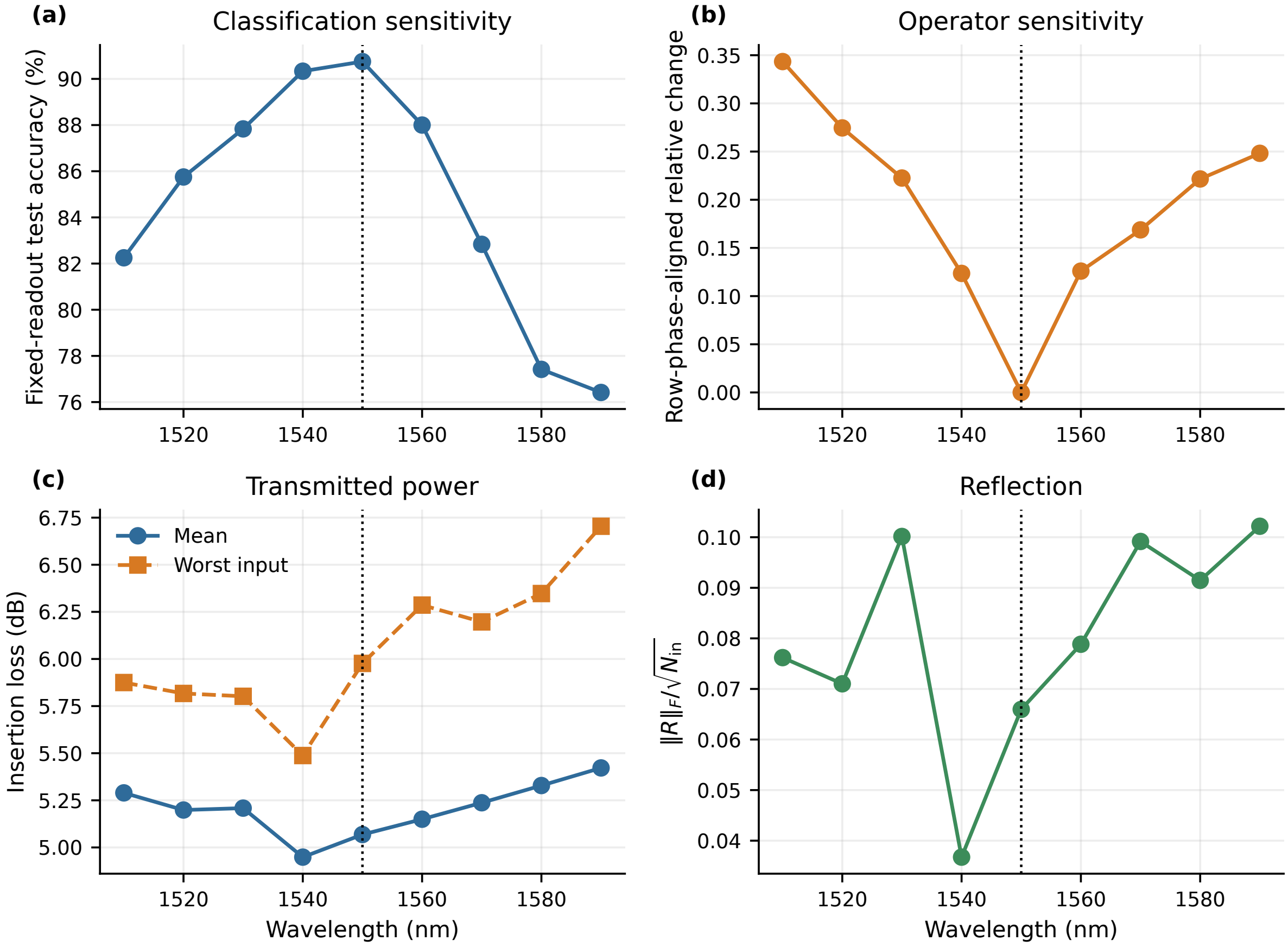

Figure S8: Fixed-readout 2D wavelength response from 1510 to 1590 nm. The vertical dotted line marks the 1550 nm design wavelength.

Figures S7 and S8 use the same binary geometry but different evaluation protocols. Figure S7 uses the stored nominal 2D response and retrains the electronic classifier for that response, giving 92.58% accuracy. Figure S8 independently simulates the geometry at each wavelength and keeps one classifier fixed across the sweep, giving 90.75% at 1550 nm.

The fabrication-variation sweep applies uniform over-etch and under-etch to the silicon pattern. Negative values denote over-etch; positive values denote under-etch; zero denotes the nominal etch. The MedNIST sweep keeps the readout fixed and uses a separately fabrication-refined design whose nominal accuracy is 96.57%. For −10 nm over-etch and +10 nm under-etch, the accuracy is 76.69% and 76.75%; at −20 and +20 nm it is 59.05% and 65.02%. Residual-slab thicknesses of 130, 140, 160, and 170 nm give 21.41%, 20.89%, 33.84%, and 12.77%, respectively. The nominal points in the connected curves use the nominal optimized responses at zero perturbation. Performance changes substantially across the tested fabrication conditions.

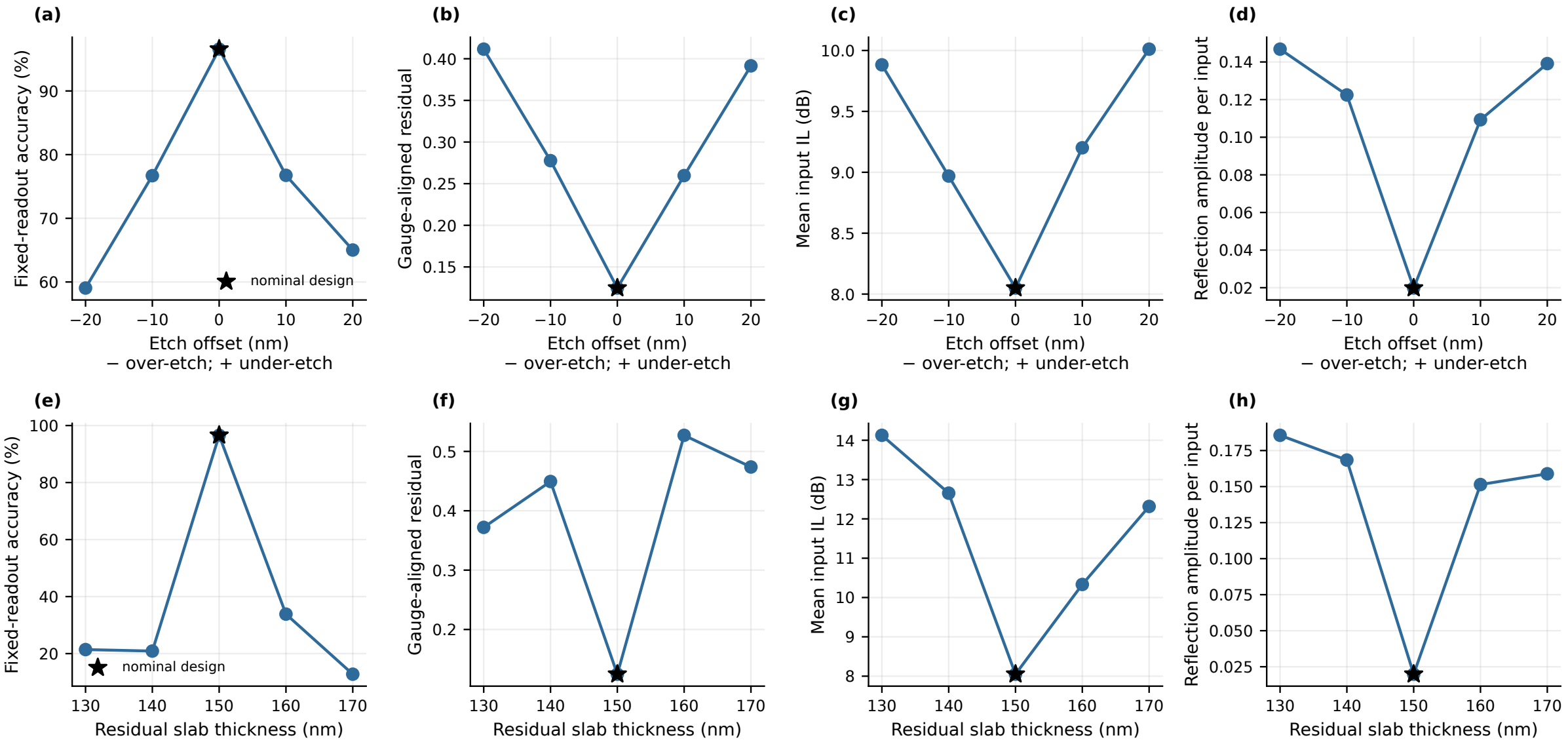

Figure S9: Fixed-readout MedNIST fabrication-tolerance sweep. The connected curves include the nominal optimized response at 0 nm etch offset and 150 nm residual slab thickness. Negative offset denotes over-etch; positive offset denotes under-etch.

The Yin–Yang etch sweep retrains the electronic classifier at every condition. Accuracy decreases from 92.58% at nominal etch to 89.08% at 20 nm over-etch and 88.00% at 20 nm under-etch.

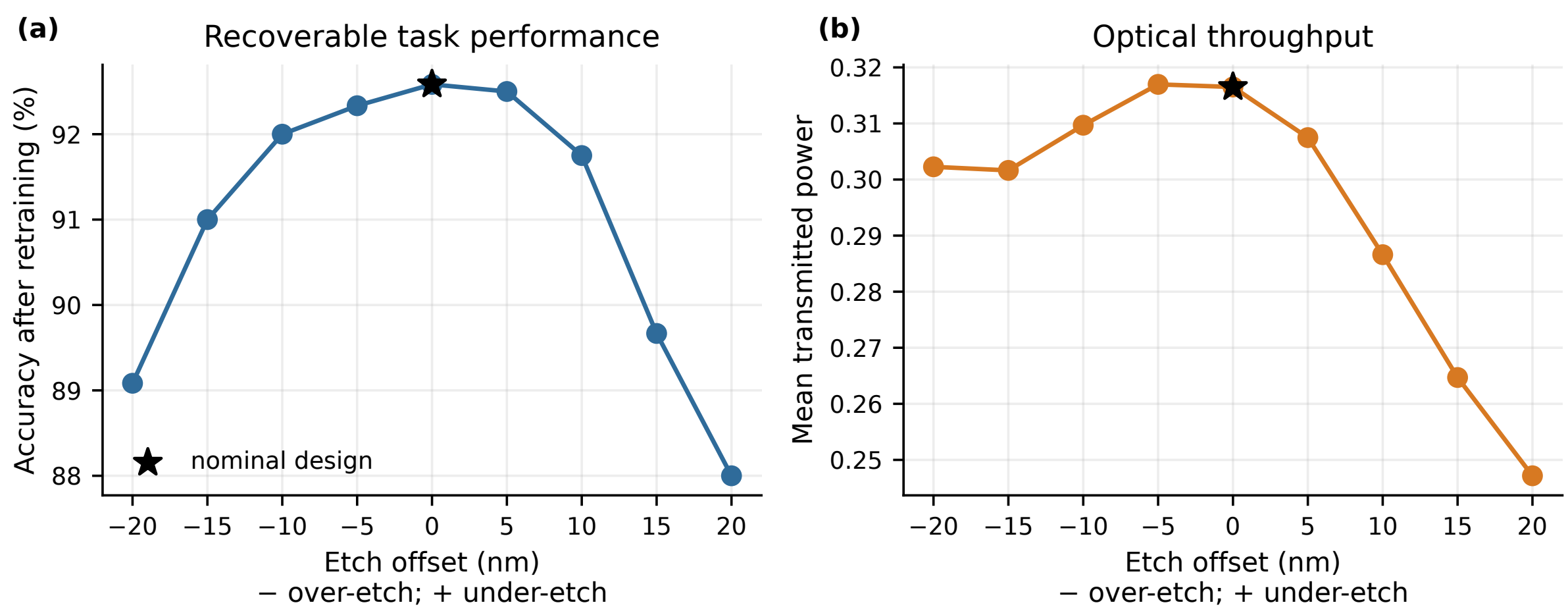

Figure S10: Yin–Yang response to uniform over-etch and under-etch, with the electronic classifier retrained at each condition. Negative offset denotes over-etch; positive offset denotes under-etch.

The input-phase study uses the seed-23 update-25 target and realized transmission matrices with the

trained readout fixed. For every encoded input component, the perturbed field is

$$a_{b,j}^{\text{noise}} = a_{b,j} \exp(i\delta\phi_{b,j}). \tag{4}$$

The phase errors are Gaussian with standard deviations $\sigma_\phi = 0°, 1°, 2°, 5°, 10°, 15°$, and $20°$. The fixed-error experiment draws one four-channel phase-error vector per trial and applies it to all 1,200 test samples. The jitter experiment draws an independent phase error for every sample and channel. Both experiments use 100 deterministic trials generated from seed 7. The optical response and readout remain fixed.

For the realized response, nominal accuracy is 93.00%. At $\sigma_\phi = 10°$, fixed per-input phase errors give $91.09 \pm 1.38\%$ mean accuracy and an 88.83% fifth percentile, while sample-dependent jitter gives $90.74 \pm 0.59\%$ and an 89.75% fifth percentile. At 20°, the corresponding values are $87.43 \pm 3.35\%$ with an 82.17% fifth percentile and $86.66 \pm 0.75\%$ with an 85.64% fifth percentile. Since the Yin–Yang phase is $-2\pi v$, a 10° phase standard deviation corresponds to a coordinate standard deviation of 0.0278.

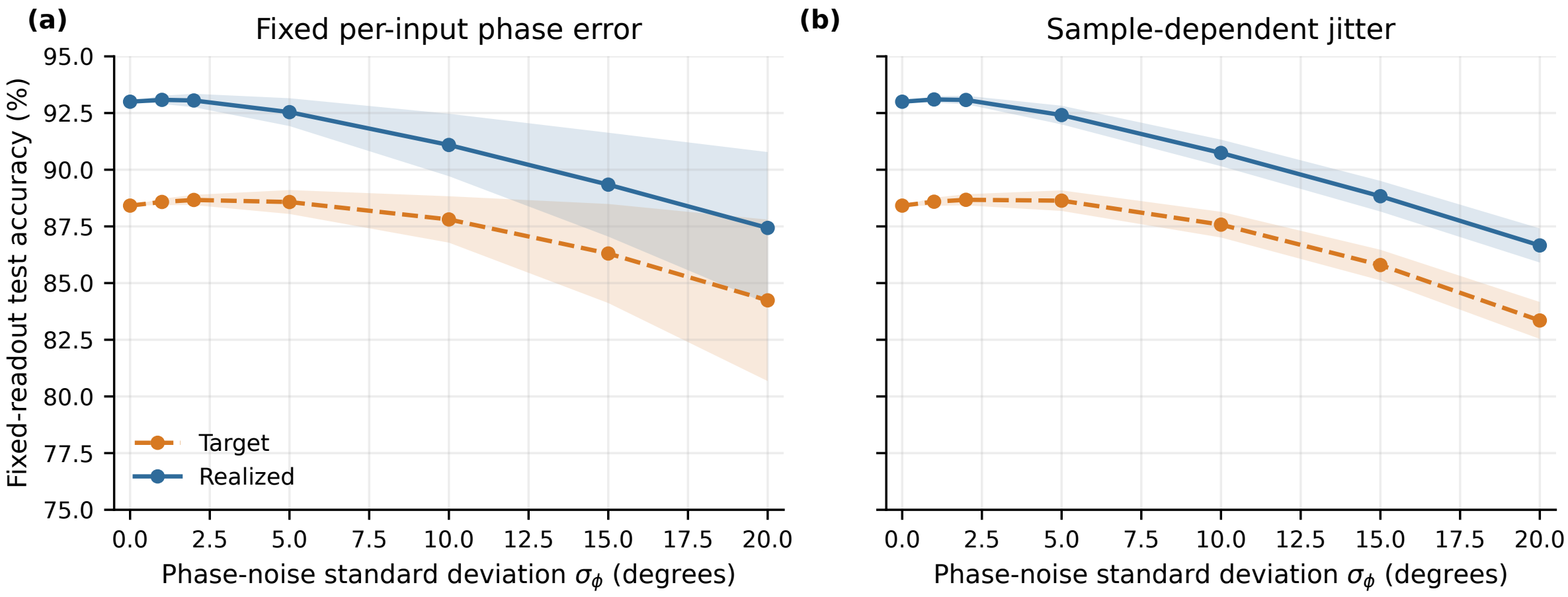

Figure S11: Yin–Yang input-phase sensitivity for the seed-23 update-25 target and realized responses. (a) One fixed phase error per input channel is applied to all test samples within a trial. (b) A new phase error is drawn for every test sample and input channel. Curves show the mean and shaded regions show one sample standard deviation across 100 deterministic Gaussian trials. The readout is fixed and each curve includes the 0° condition.
